# tRF-3005a regulates exon skipping of SPAG4 by interacting with RALY to drive gastric cancer progression

**DOI:** 10.1038/s41420-026-03049-3

**Published:** 2026-03-24

**Authors:** Huaiping Cui, Yancong Yuan, Yizhe Yin, Ruihong Gao, Zhaodong Liu, Lipan Peng, Jinshen Wang, Zhu Wang, Tingting Song, Jinglei Liu

**Affiliations:** 1 Department of Gastrointestinal Surgery, Shandong Provincial Hospital Affiliated to Shandong First Medical University, Jinan, Shandong China; 2 Department of Gastrointestinal Surgery, Shandong Provincial Hospital, Cheeloo College of Medicine, Shandong University, Jinan, Shandong China; 3 Shandong Provincial Laboratory of Translational Medicine Engineering for Digestive Tumors, Shandong Provincial Hospital, Jinan, Shandong China; 4 Department of Gastrointestinal Surgery, The Third Affiliated Hospital of Shandong First Medical University (Affiliated Hospital of Shandong Academy of Medical Sciences), Jinan, Shandong China; 5 Department of Radiotherapy, Linyi Cancer Hospital, Linyi, Shandong China

**Keywords:** Cancer, Gastrointestinal diseases

## Abstract

Transfer RNA-derived fragments (tRFs) are emerging regulators in cancer, yet their role in the development and progression of gastric cancer (GC) remains unclear. Through RNA sequencing technology, this study identified a tRNA-derived fragment, tRF-3005a, that is significantly upregulated in GC tissues and cell lines and is associated with poor prognosis. Functionally, it promotes the proliferation, migration, and invasion of GC cells. Mechanistically, tRF-3005a bound to RALY, enhancing its interaction with SPAG4 mRNA, suppressing exon 8 skipping and leading to an increased generation of oncogenic SPAG4-L isoforms, thereby activating GRB14/PI3K/AKT signaling and ultimately promoting GC progression. This study reveals a novel mechanism wherein tRF-3005a promotes gastric cancer development by regulating RALY-mediated alternative splicing of SPAG4 to activate the GRB14/PI3K/AKT pathway, suggesting it may serve as a prognostic biomarker and therapeutic target.

## Introduction

Gastric cancer (GC) is one of the most common malignant tumors worldwide. It ranks as the 5th most frequently diagnosed malignancy and simultaneously the 3rd leading cause of cancer-related death, imposing a heavy burden on healthcare systems across the globe, with the burden being particularly prominent in East Asia [1]. Due to the subtlety of early-stage symptoms and inadequate screening measures, the majority of patients are diagnosed at an advanced stage [2, 3]. Despite significant advancements in cancer treatment strategies, the prognosis for gastric cancer (GC) patients remains poor [4, 5]. Therefore, there is an urgent need to identify novel biomarkers and conduct in-depth investigations into their mechanisms of action, so as to improve early screening, treatment efficacy, and prognosis of GC.

In recent years, researchers have identified a large number of small noncoding RNAs (sncRNAs) with regulatory functions [6–8]. A growing body of research indicates that sncRNAs are dysregulated in gastric cancer (GC), positioning them as promising biomarkers for early diagnosis, therapeutic targets, and prognostic evaluation indicators [9–11]. With the advancement of high-throughput sequencing and microarray technologies, a novel class of tRNA-derived fragments (tRFs and tiRNAs)—categorized under the umbrella of sncRNAs—has emerged as a new focus in cancer research [12, 13]. Numerous studies have confirmed that tRFs and tiRNAs extensively influence the progression of various cancers, exerting their effects through direct binding to RNAs or proteins [14]. Our team is among the earliest to conduct research on tRFs: in our previous studies, we found that a novel 3′tRNA-derived fragment, tRF-Val, can promote tumor cell proliferation and inhibit apoptosis in GC by binding to EEF1A1 [15]; another novel 5′tRNA-derived fragment, tRF-Tyr, exerts a tumor-suppressive effect on progression by binding to hnRNPD [16]. Similar findings have been reported in other studies; for instance, tRF-33 inhibits GC progression by binding to AGO2 [17], and tRF-27 enhances trastuzumab resistance in human epidermal growth factor receptor 2 (HER2)-positive breast cancer through competitive binding to G3BPs [18].

Alternative splicing (AS) is a crucial post-transcriptional regulatory mechanism that generates multiple transcripts from a single gene, thereby increasing transcriptome complexity and protein diversity [19]. Aberrant AS events are commonly associated with various diseases, with a particular prominence in cancer [20, 21]. Multiple studies have confirmed that AS events in tumor tissues exhibit significant alterations compared to those in normal tissues [22–24]. Typically, the occurrence of AS relies on the regulation of splicing factors [25, 26]. Heterogeneous nuclear ribonucleoproteins (hnRNPs) are a class of RNA-binding proteins that can function as splicing factors [27, 28]. For instance, hnRNP RALY (a member of the hnRNP family, abbreviated as RALY) is involved in AS events and promotes tumor progression [26, 29]. However, current research on RALY in gastric cancer (GC) remains relatively limited, which provides a new direction for mechanistic studies in this field.

In this study, high-throughput sequencing revealed that a novel tRNA-derived fragment, tRF-3005a, is significantly upregulated in gastric cancer (GC) tissues, suggesting its potential involvement in the underlying tumorigenic mechanisms of GC. Further investigations demonstrated that tRF-3005a directly interacts with RALY, modulating RALY-mediated alternative splicing of exon 8 of the SPAG4 gene, and consequently influencing tumor progression. Our findings uncover the crucial role of the tRF-3005a–RALY regulatory axis in GC and establish a link between alternative splicing (AS) and tumor progression, providing new insights for future research.

## Results

### tRF-3005a is upregulated in gastric cancer and predicts poor prognosis

**Fig. 1:**
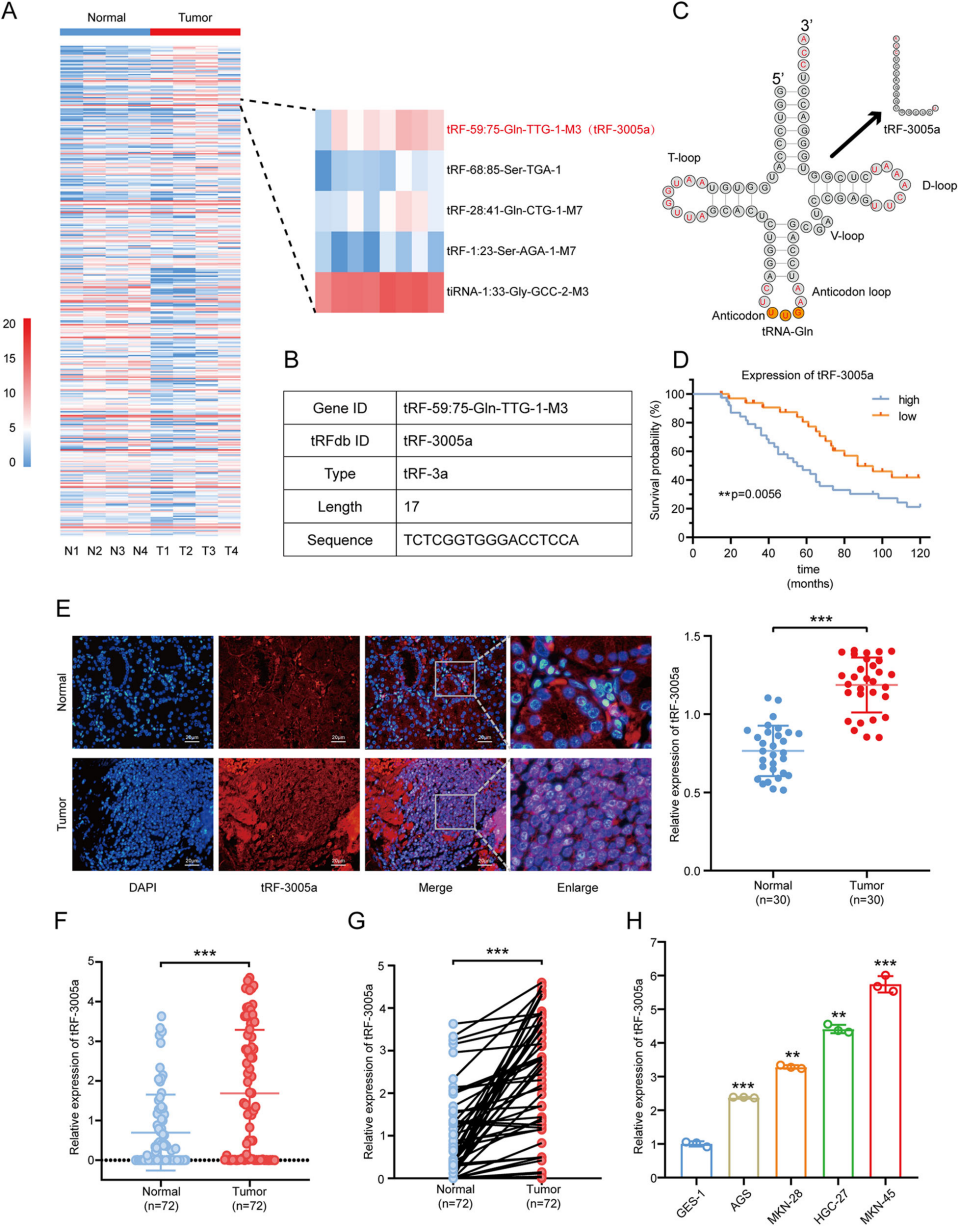
tRF-3005a is significantly upregulated in GC. **A** Heatmap of tRFs and tiRNAs in tumor and normal groups. **B** Basic information of tRF-3005a. **C** Location and sequence of tRF-3005a in tRNA-3005a. **D** Overall survival (OS) analysis of the tRF-3005a in 72 GC patients. **E** Localization and expression of tRF-3005a in GC tissue and normal tissue were detected by FISH assays. **F**, **G** The relative expression of tRF-3005a in 72 GC tissues and paired normal tissues was detected by qRT-PCR. **H** The relative expression of tRF-3005a in GC cell lines and GES-1 were detected by qRT-PCR. Data were expressed as mean ± SD. (Student′s *t*-test; Mann‒Whitney *U*-test; Paired *t*-test; Log-rank test; *P < 0.05; **P < 0.01; and ***P < 0.001). N means normal tissues, T means tumor tissues.

To explore tRFs and tiRNAs potentially involved in the progression of gastric cancer, we referred to previous studies [15] and identified that tRF-3a was upregulated in GC, indicating its close association with the disease. Furthermore, high-throughput sequencing of four pairs of GC tissues and adjacent normal tissues confirmed the upregulation of tRFs of the 3a type in GC, with tRF-59:75-Gln-TTG-1-M3 (tRF-3005a) being significantly elevated. Based on these findings, we selected tRF-3005a for subsequent investigations (Fig. 1A). The basic information of tRF-3005a and its location in tRNA-3005a were obtained from MINTbase (Fig. 1B, C, https://cm.jefferson.edu/MINTbase) [30]. The Kaplan–Meier survival analysis demonstrated that higher levels of tRF-3005a were notably associated with poorer overall survival (OS) in a cohort of 70 GC patients (Fig. 1D). To examine the subcellular localization of tRF-3005a, a FISH assay was carried out on 30 GC tissues and adjacent paired normal tissues. The results showed a markedly higher fluorescence intensity in the tumor tissues compared to the normal ones, and tRF-3005a was mainly localized in the cell nucleus (Fig. 1E). These results suggested that tRF-3005a was upregulated in tumor tissues compared to normal tissues, and that molecular nuclear localization was linked to functions such as transcription regulation and RNA processing [31, 32]. In addition, tRF-3005a expression was analyzed in 72 GC and normal tissues by qRT-PCR, showing considerably higher levels in GC tissues (Fig. 1F, G). Correlation analysis revealed that elevated tRF-3005a expression was correlated with larger tumor size, deeper invasion, and an increased likelihood of lymph node metastasis in gastric cancer tissues (Table 1). Moreover, tRF-3005a expression was elevated in GC cell lines compared to the GES-1 cell lines (Fig. 1H). The GC cell with the highest (MKN-45) and lowest (AGS) expression was selected for functional and mechanism studies.

**Table 1:** Correlation between tRF-3005a expression and clinicopathological characteristics in 72 GC patients.

Parameters	Cases	tRF-3005a expression		*p*-value
		Low	High	
Total	72	35	37	
Age				0.221
<60	22	14	8	
≥60	50	24	26	
Gender				0.836
Male	40	19	21	
Female	32	16	16	
Tumor invasion				0.014^*^
T1–T2	27	17	10	
T3–T4	45	15	30	
Lymph node metastasis				0.045^*^
N0	24	15	9	
N1-N3	48	18	30	
TNM stage				0.355
I–II	35	15	20	
III	37	25	22	
Tumor size				0.002^**^
<5 cm	36	26	10	
≥5 cm	36	12	24	

^Statistically significant: *p < 0.05 and **p < 0.01.^

### tRF-3005a is localized in the nucleus and facilitates GC cells' proliferation, invasion, and migration

**Fig. 2:**
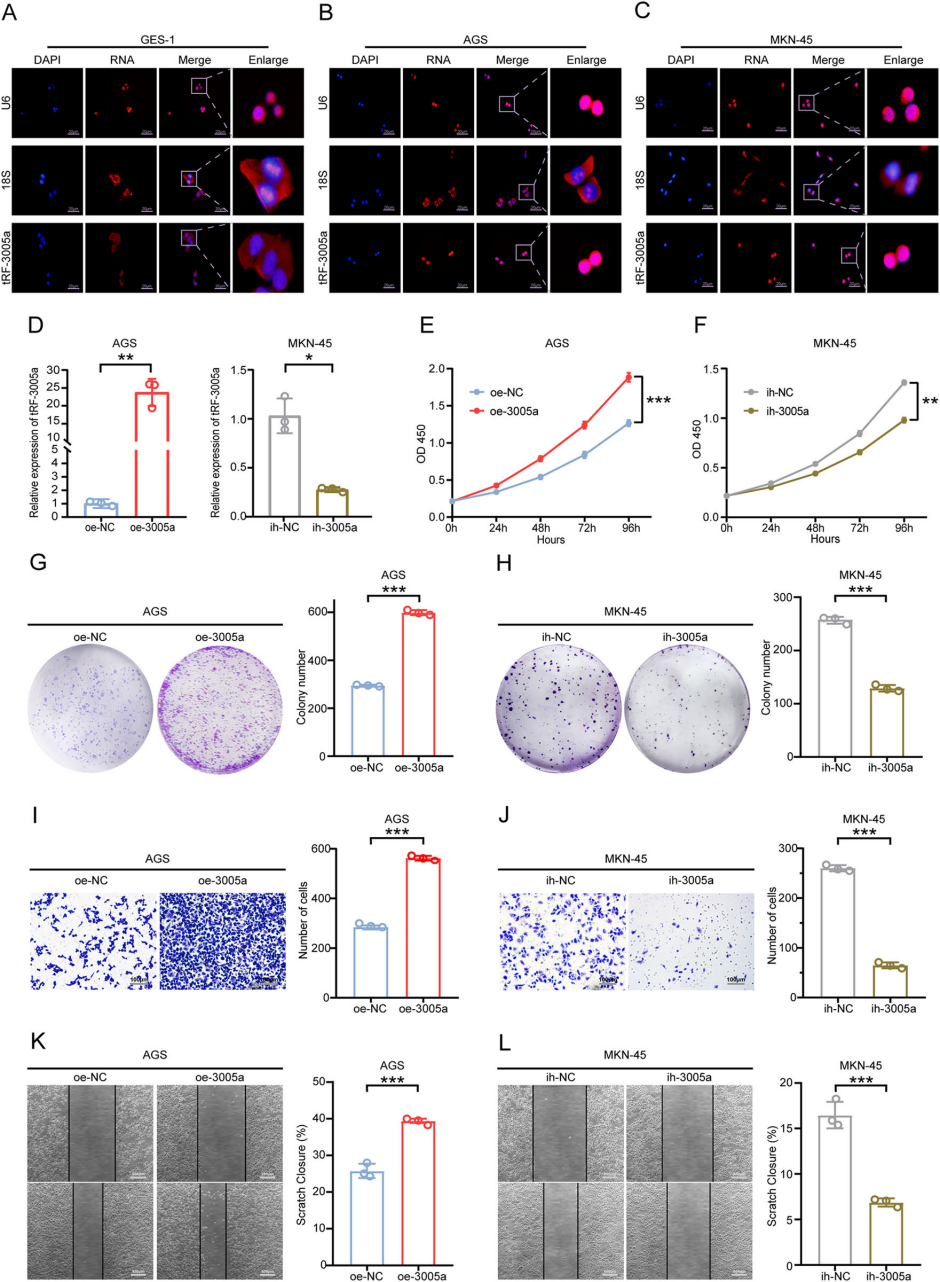
Nuclear tRF-3005a promotes proliferation, invasion, and migration of GC cells in vitro. **A**–**C** The FISH assay indicated that compared with GES-1 cells, tRF-3005a was mainly localized in the nucleus of GC cells. **D** The overexpression efficiency of tRF-3005a in AGS cells and the inhibition efficiency in MKN-45 cells were verified by qRT-PCR. CCK-8 assays were performed to detect the proliferation of AGS cells transfected with oe-NC or oe-3005a (**E**), and MKN-45 cells transfected with ih-NC or ih-3005a (**F**). Colony formation assays were performed to detect the proliferation of AGS cells transfected with oe-NC or oe-3005a (**G**), and MKN-45 cells transfected with ih-NC or ih-3005a (**H**). Transwell assays were performed to detect the invasion of AGS cells transfected with oe-NC or oe-3005a (**I**), and MKN-45 cells transfected with ih-NC or ih-3005a (**J**). Scratch healing assays were performed to detect the migration of AGS cells transfected with oe-NC or oe-3005a (**K**), and MKN-45 cells transfected with ih-NC or ih-3005a (**L**). Data were expressed as mean ± SD. (Student′s *t*-test; *P < 0.05; **P < 0.01, and ***P < 0.001). OD450 means optical density at 450 nm, oe means overexpression, ih means inhibition, NC means negative control, and 3005a means tRF-3005a.

In order to further validate the subcellular localization of tRF-3005a, a FISH assay was carried out in GC cell lines as well as the normal gastric epithelial cell line GES-1. The findings showed tRF-3005a was predominantly nuclear in GC cells (AGS, MKN-45 cells), but cytoplasmic in GES-1 cells (Fig. 2A–C). Based on the relative expression levels of tRF-3005a, overexpression and inhibition models of tRF-3005a were established in AGS and MKN-45 cells to further explore its biological function in GC. The efficiencies of tRF-3005a overexpression and inhibition in both cell lines were confirmed by qRT-PCR (Fig. 2D). CCK-8 assay demonstrated that tRF-3005a overexpression markedly enhanced the proliferation of AGS and GES-1 cells, whereas its inhibition significantly suppressed MKN-45, AGS, HGC-27, and MKN-28 cells' proliferation (Fig. 2E, F; Fig. S1A–D). According to the colony formation assay, tRF-3005a overexpression substantially increased colony formation in AGS and GES-1 cells, while its silencing significantly impaired this ability in MKN-45, AGS, HGC-27, and MKN-28 cells (Fig. 2G, H; Fig. S1E–H). Transwell invasion assay indicated that tRF-3005a overexpression increased the invasive capacity of AGS cells, whereas its knockdown reduced their invasive ability (Fig. 2I, J). Moreover, scratch assays showed that tRF-3005a overexpression increased migration in AGS, while its inhibition reduced migration in MKN-45(Fig. 2K, L). Overall, tRF-3005a facilitated GC cell proliferation, invasion, and migration in vitro.

### tRF-3005a promotes GC progression through its direct binding to RALY, and is widely involved in alternative splicing

**Fig. 3:**
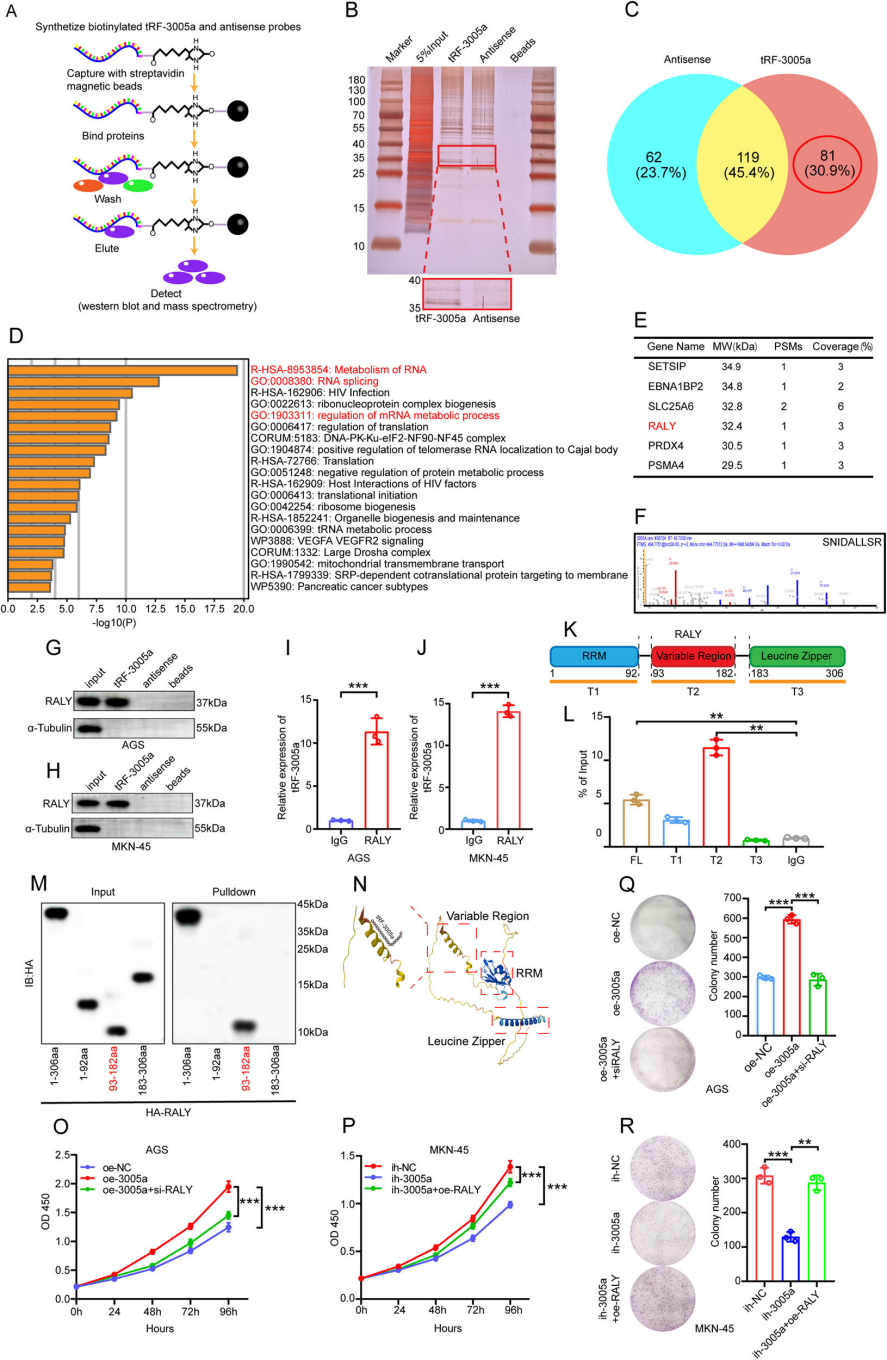
tRF-3005a promotes GC progression through direct binding to RALY. **A** The workflow of the tRF-3005a pulldown assay. **B** Silver staining assay was performed to detect the differential proteins obtained by the pulldown assay in MKN-45 cells. **C** Mass spectrometry identified 81 specific binding proteins. **D** GO pathway analysis of 81 tRF-3005a-interacting proteins derived from mass spectrometry. **E** Silver staining combined with GO analysis identified RALY as the specific binding protein. **F** The specific peptide of RALY identified by mass spectrometry. **G**, **H** Independent RNA-pulldown assays and WB assays confirmed the interaction of tRF-3005a and RALY in AGS and MKN-45 cells, respectively. **I**, **J** RIP assays were performed to detect the binding of tRF-3005a and RALY in AGS and MKN-45 cells, respectively. **K** Schematic of RALY proteins and three truncated mutants (T1: 1–92; T2: 93–182; T3: 183–306). **L** RIP assays were conducted with anti-HA antibodies in MKN-45 cells transfected with plasmids encoding the HA-tagged FL and truncated mutants (T1–T3) of RALY. **M** Biotin-labeled tRF-3005a was incubated with HA-tagged full-length or truncated RALY, pulled down by streptavidin beads, and detected by Western blotting. **N** Schematic of the three domains of RALY and the tRF-3005a binding site on RALY. CCK-8 rescue assays were performed to detect the proliferation of AGS cells transfected with oe-NC, oe-3005a, or oe-3005a+si-RALY (**O**), and MKN-45 cells transfected with ih-NC, ih-3005a, or ih-3005a+oe-RALY (**P**). Colony formation rescue assays were performed to detect the proliferation of AGS cells transfected with oe-NC, oe-3005a, or oe-3005a+si-RALY (**Q**), and MKN-45 cells transfected with ih-NC, ih-3005a, or ih-3005a+oe-RALY (**R**). Data were expressed as mean ± SD. (Student′s *t*-test; *P < 0.05; **P < 0.01, and ***P < 0.001). MW means Molecular Weight, PSMs means peptide-spectrum matches, FL means full-length, RRM means RNA recognition motif, IB means immunoblot, and si means small interfering RNA.

Previous studies showed that tRF-3005a is mainly nuclear, and tRFs can exert biological functions as sncRNAs by binding proteins or RNAs [12]. Therefore, we hypothesized that tRF-3005a may interact with nuclear proteins to exert biological effects. To test this, RNA pull-down was performed in MKN-45 cells, which shows the highest tRF-3005a expression (Fig. 3A). Biotin-labeled tRF-3005a and antisense probes were incubated with cell lysates, and silver staining revealed a specific band at 35–40 kDa (Fig. 3B). Mass spectrometry identified 81 specific binding proteins (Table S5) absent in the control, which were further analyzed by GO pathway enrichment using Metascape (https://metascape.org, Fig. 3C, D) [33]. The results revealed numerous partners linked to RNA splicing, indicating that tRF-3005a is broadly involved in alternative splicing. Silver staining combined with GO analysis ultimately identified RALY as a specific binding partner (Fig. 3E, F). RNA pull-down and Western blot confirmed the tRF-3005a–RALY interaction in AGS and MKN-45 cells (Fig. 3G, H), further supported by RIP assays showing enrichment of tRF-3005a with RALY antibody compared to IgG control (Fig. 3I, J). RALY, localized in the nucleus as a splicing factor, regulates alternative splicing of downstream target genes [34]. It is composed of an RNA recognition motif (RRM), a variable region, and a Leucine Zipper domain (Fig. 3K). Based on this structural organization, we constructed three HA-tagged RALY fragments to identify the specific binding region between tRF-3005 and RALY. RIP assays showed that tRF-3005a mainly bound the variable region (93–182aa) of RALY (Fig. 3L). RNA pull-down further confirmed this, as tRF-3005a pulled down only full-length RALY and fragments containing the variable region (Fig. 3M). Through the AlphaFold Protein Structure Database, we obtained the three domains of RALY and predicted the interaction region with tRF-3005a (Fig. 3N, https://www.alphafold.ebi.ac.uk) [35]. The variable region of the protein, serving as a functional domain, has been suggested to undergo conformational changes upon interaction with RNA, thereby potentially altering the protein’s biological function [36–38]. To further explore their functions, WB and qRT-PCR analyses indicated that tRF-3005a modulation had no significant effect on the total levels of RALY protein or mRNA (Fig. S2A–D). Additionally, altering RALY levels in AGS and MKN-45 cells did not affect tRF-3005a expression (Fig. S2E, F). CCK-8 and colony formation rescue assays showed that RALY knockdown or overexpression reversed the effects of tRF-3005a in AGS and MKN-45 cells (Fig. 3O–R). These results indicate the role of the tRF-3005a–RALY axis in GC cell growth. Taken together, we hypothesize that tRF-3005a may potentially induce conformational changes in RALY by binding to its variable region, thereby regulating RALY-mediated alternative splicing of downstream targets and promoting gastric cancer progression.

### RALY is upregulated and promotes growth in GC cells

TCGA database analysis revealed that RALY is highly upregulated in a variety of human cancers, including gastric cancer (http://timer.cistrome.org, Fig. S3A) [39]. OS analysis further revealed that elevated RALY expression correlates with poor prognosis in GC patients (https://kmplot.com, Fig. S3B) [40]. To investigate the role of RALY in gastric cancer, IHC staining of 30 GC tissues and paired normal tissues showed higher RALY expression in tumors (Fig. S3C). Western blot analysis confirmed elevated RALY levels in GC cell lines (MKN-45, MKN-28, HGC, AGS) compared with GES-1, as well as in paired GC tissues relative to adjacent normal tissues (Fig. S3D, E). Furthermore, RALY overexpression and knockdown models in AGS and MKN-45 cells showed that overexpression enhanced, whereas knockdown suppressed, gastric cancer cell proliferation in CCK-8 and colony formation assays (Fig. S3F–H). Similar results were observed upon RALY overexpression in GES-1 cells (Fig. S3I, J). Thus, RALY promotes gastric cancer progression.

### RALY promotes GC Cells proliferation by inhibiting SPAG4 exon skipping

**Fig. 4:**
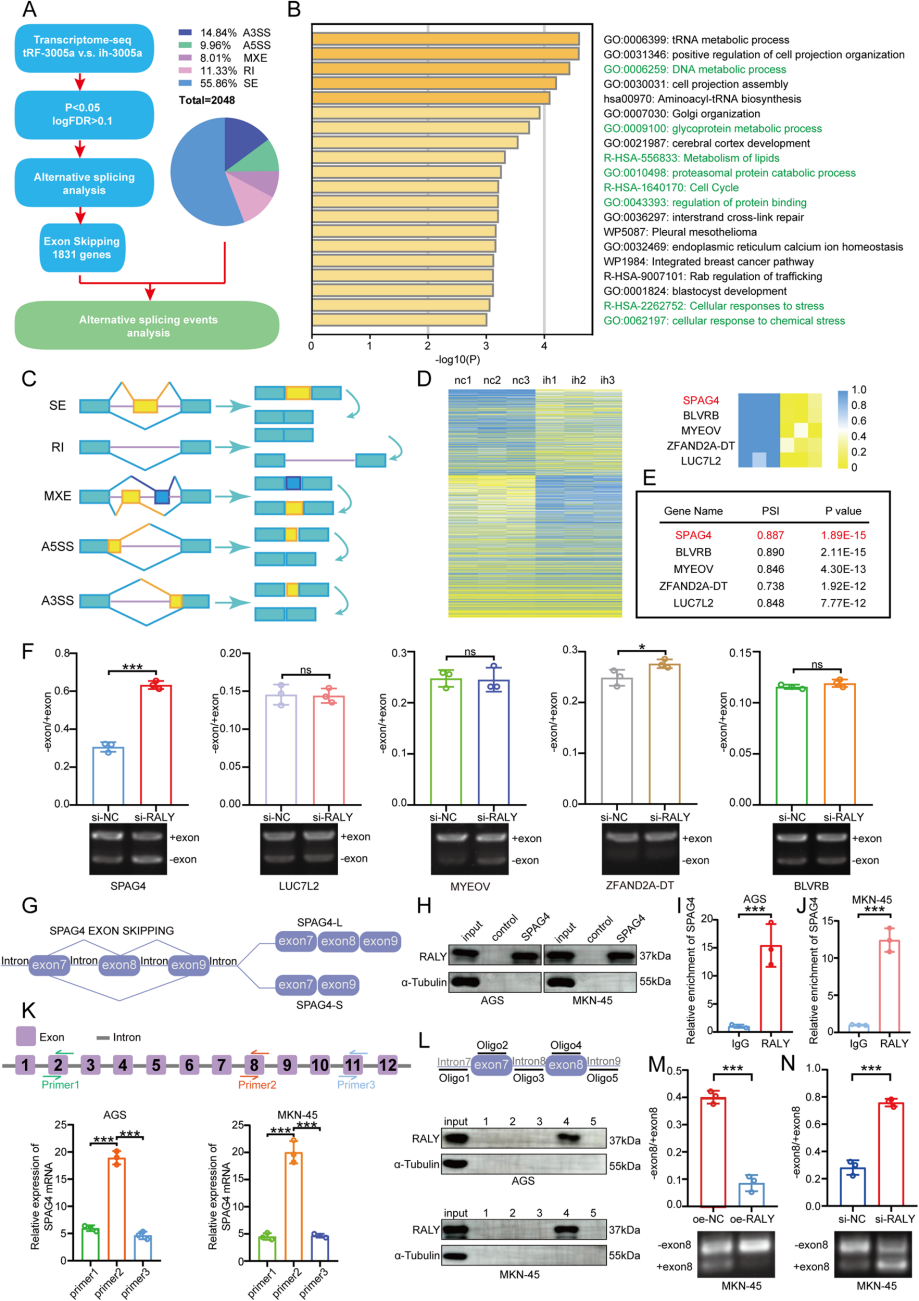
RALY promotes GC Cells proliferation by inhibiting SPAG4 exon skipping. **A** Schematic workflow of RNA-seq and alternative splicing events analysis. **B** GO pathway analysis via the Metascape program of 1831 genes with altered exon skipping derived from RNA-seq. **C** Schematic diagram of different types of alternative splicing events: exon skipping (ES), intron retention (IR), mutually exclusive exons (MXE), alternative 5′ splice site (A5SS), and alternative 3′ splice site (A3SS). **D** Heatmap of gene expression changes following tRF-3005a inhibition in MKN-45 cells. **E** SPAG4 exhibited the most significant *p*-value among the five candidate genes. **F** qRT-PCR assays were conducted to show the ratio of candidate genes exon skipping in MKN-45 cells transfected with si-NC and si-RALY. **G** Schematic representation of alternative splicing patterns in SPAG4. **H** RNA pull-down assays were performed to confirm the interaction between RALY and SPAG4 mRNA. **I**, **J** RIP assays further validated this interaction between RALY and SPAG4 mRNA. **K** Design of three specific primers (primers 1–3), and RIP assays were performed to detect the specific interaction site between RALY and SPAG4. **L** A series of bait-oligos was constructed, and RNA pull-down assays were performed to identify the site of SPAG4. **M**, **N** qRT-PCR assays were conducted to show the ratio of SPAG4-S/SPAG4-L in MKN-45 cells transfected with oe-NC, oe-RALY, si-NC, and si-RALY. Data were expressed as mean ± SD. (Student′s *t*-test, ***P < 0.001). NC means negative control, ih means ih-3005a.

The above studies have confirmed that RALY could promote the growth of GC cells, but the specific mechanism is still not clear. To identify downstream target genes of RALY regulated by tRF-3005a (tRF-3005a–RALY axis), high-throughput mRNA sequencing (RNA-seq) was performed on tRF-3005a inhibition cells (MKN-45). Analysis of alternative splicing events revealed that exon skipping was the most affected (Fig. 4A). Subsequently, genes with altered exon skipping were subjected to GO analysis. The results indicated that the affected genes were rich in cellular processes. (Fig. 4B). The figure (Fig. 4C) illustrates AS: exon skipping (ES), intron retention (IR), mutually exclusive exons (MXE), alternative 5′ splice site (A5SS), and alternative 3′ splice site (A3SS). The heatmap displays the fold changes of exon skipping genes (Fig. 4D). Based on the significance of the changes, we ranked the genes identified by sequencing, and SPAG4 showed the highest significance (Fig. 4E). Following RALY knockdown, qRT-PCR analysis of candidate genes revealed that only SPAG4 exhibited a significant increase in the -exon/+exon ratio (Fig. 4F). Ultimately, SPAG4 was selected as the downstream target gene of RALY. Its splicing pattern was depicted via the TCGA SpliceSeq Database (https://bioinformatics.mdanderson.org/TCGASpliceSeq/singlegene.jsp, Fig. 4G) [41]. RNA pull-down using a biotin-labeled SPAG4 probe verified the interaction between RALY and SPAG4 mRNA (Fig. 4H). Briefly, biotin-labeled SPAG4 and control probes were incubated with cell lysates, and the pulled-down complexes were analyzed by Western blotting to detect RALY. As a result, RALY was readily detected in the SPAG4 probe pull-down but not in the control group, supporting a specific interaction between RALY and SPAG4 mRNA. In addition, RIP assays further supported this interaction (Fig. 4I, J). Next, three primers (primer 1–3) were designed to map the precise interaction regions between RALY and SPAG4. RIP assays showed that RALY was found to bind mainly to exon 8 of SPAG4 (Fig. 4K). To further validate the RIP results, we designed a set of bait RNAs for RNA-pulldown assays and found that oligo 4 of SPAG4 showed the unique affinity for RALY (Fig. 4L, Fig. S4A). We next investigated the role of RALY in regulating the alternative splicing of SPAG4 exon 8 in GC cells. The result showed that in MKN-45 and AGS cells, RALY silencing promoted exon 8 skipping, whereas RALY overexpression exerted the opposite effect (Fig. 4M, N; Fig. S4B, C). Correlation analysis of RALY mRNA expression and the SPAG4-S/SPAG4-L (−exon 8/+exon 8) ratio in gastric cancer tissues by qRT-PCR revealed that increasing RALY expression was associated with a progressive decline in the ratio, further indicating that RALY suppresses exon 8 skipping of SPAG4 (Fig. S4D). To delineate the contribution of SPAG4 alternative splicing to GC progression, we constructed GC cells overexpressing SPAG4-L and SPAG4-S. CCK-8 and colony formation assays revealed that SPAG4-L, but not SPAG4-S, promoted tumor cell growth (Fig. S4E–H). In rescue experiments, the proliferation and colony formation defects induced by RALY knockdown were restored by overexpression of SPAG4-L, whereas SPAG4-L silencing abrogated the growth-promoting effects of RALY overexpression (Fig. S4I–L). Collectively, RALY targets SPAG4 exon 8 to inhibit its skipping, promoting GC cells' growth.

### tRF-3005a facilitates GC progression by strengthening RALY-dependent repression of SPAG4 exon skipping

**Fig. 5:**
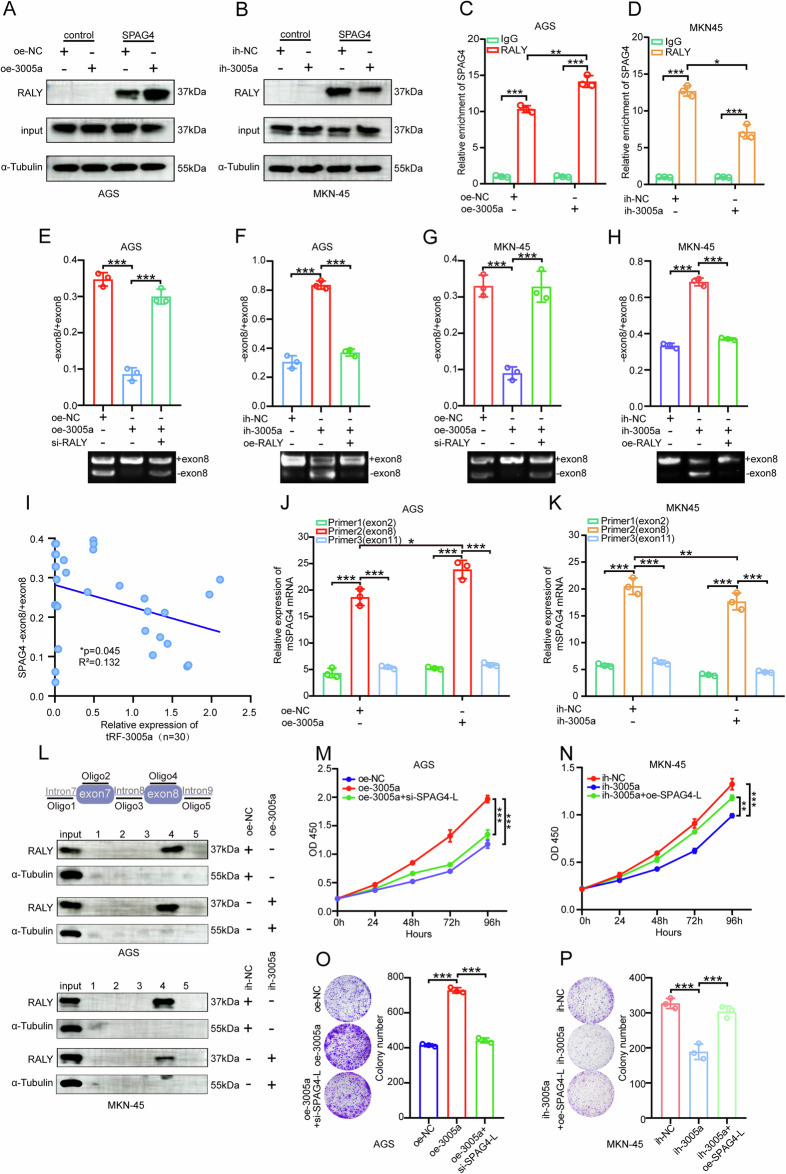
tRF-3005a promotes the RALY-dependent repression of SPAG4 exon skipping. **A**, **B** RNA pull-down assays were performed to detect the interaction between RALY and SPAG4 in AGS cells transfected with oe-NC or oe-3005a and MKN-45 cells transfected with ih-NC or ih-3005a. **C**, **D** RIP assays were performed to detect the interaction between RALY and SPAG4 in AGS cells transfected with oe-NC or oe-3005a and MKN-45 cells transfected with ih-NC or ih-3005a. qRT-PCR rescue assays were conducted to show the ratio of SPAG4-S/SPAG4-L in AGS and MKN-45 cells transfected with oe-NC, oe-3005a, or oe-3005a+si-RALY (**E**, **G**); ih-NC, ih-3005a, or ih-3005a+oe-RALY (**F**, **H**). **I** Correlation analysis of tRF-3005a expression level and the ratio of SPAG4-S/L in 30 GC tissues by qRT-PCR. **J**–**L** RIP and RNA pull-down assays were performed to detect the interaction between RALY and the exon of SPAG4 in AGS cells transfected with oe-NC or oe-3005a, and MKN-45 cells transfected with ih-NC and ih-3005a. CCK-8 rescue and colony formation rescue assays were performed to detect the proliferation of AGS cells transfected with oe-NC, oe-3005a, or oe-3005a+si-SPAG4-L (**M**, **O**), and MKN-45 cells transfected with ih-NC, ih-RALY, or ih-RALY+oe-SPAG4-L (**N**, **P**). Data were expressed as mean ± SD. (Student′s *t*-test, Pearson correlation test with two-tailed; *P < 0.05; **P < 0.01, and ***P < 0.001).

The above experiments suggest that tRF-3005a binds to the variable region of RALY and may potentially induce conformational changes, and that RALY was capable of suppressing SPAG4 exon 8 skipping. To comprehensively investigate whether tRF-3005a binding and the resulting conformational changes in RALY (tRF-3005a–RALY axis) affect SPAG4 alternative splicing, RNA-pulldown and RIP assays were performed in cell lines with tRF-3005a knockdown and overexpression, respectively (Fig. 5A–D). The results indicated that overexpression of tRF-3005a enhanced the binding and interaction between RALY and SPAG4, whereas knockdown of tRF-3005a had the opposite effect. We next examined RALY’s role in tRF-3005a–regulated SPAG4 exon 8 splicing. The results showed that overexpression of tRF-3005a suppressed SPAG4 exon 8 skipping, whereas this inhibition was reversed by RALY knockdown. Conversely, the promoting effect on exon 8 skipping caused by tRF-3005a inhibition could be counteracted by RALY overexpression. These results indicate that the regulatory effect of tRF-3005a on SPAG4 exon 8 splicing depends on RALY. Correlation analysis in GC tissues revealed that higher tRF-3005a expression corresponds to a lower SPAG4-S/SPAG4-L ratio, indicating its role in suppressing SPAG4 exon 8 skipping (Fig. 5I). RIP experiments using three primers (Fig. 5J, K), together with oligonucleotide pulldown assays (Fig. 5L), further confirmed that tRF-3005a promotes the specific binding of RALY to SPAG4 exon 8. The above experiments indicate that the tRF-3005a–RALY axis affects SPAG4 alternative splicing. Furthermore, CCK-8 and colony formation rescue assays demonstrated that the proliferative effect of tRF-3005a overexpression in AGS was reversed upon SPAG4-L knockdown, and overexpression of SPAG4-L had the opposite outcome in MKN-45 cells (Fig. 5M–P). Collectively, we conclude that tRF-3005a suppresses SPAG4 exon 8 skipping in a RALY-dependent manner and further amplifies RALY’s inhibitory effect on SPAG4 exon 8 skipping. Thus, the tRF-3005a–RALY axis promotes gastric cancer progression by inhibiting SPAG4 exon 8 skipping.

### tRF-3005a–RALY regulates the GRB14/PI3K/AKT pathway

**Fig. 6:**
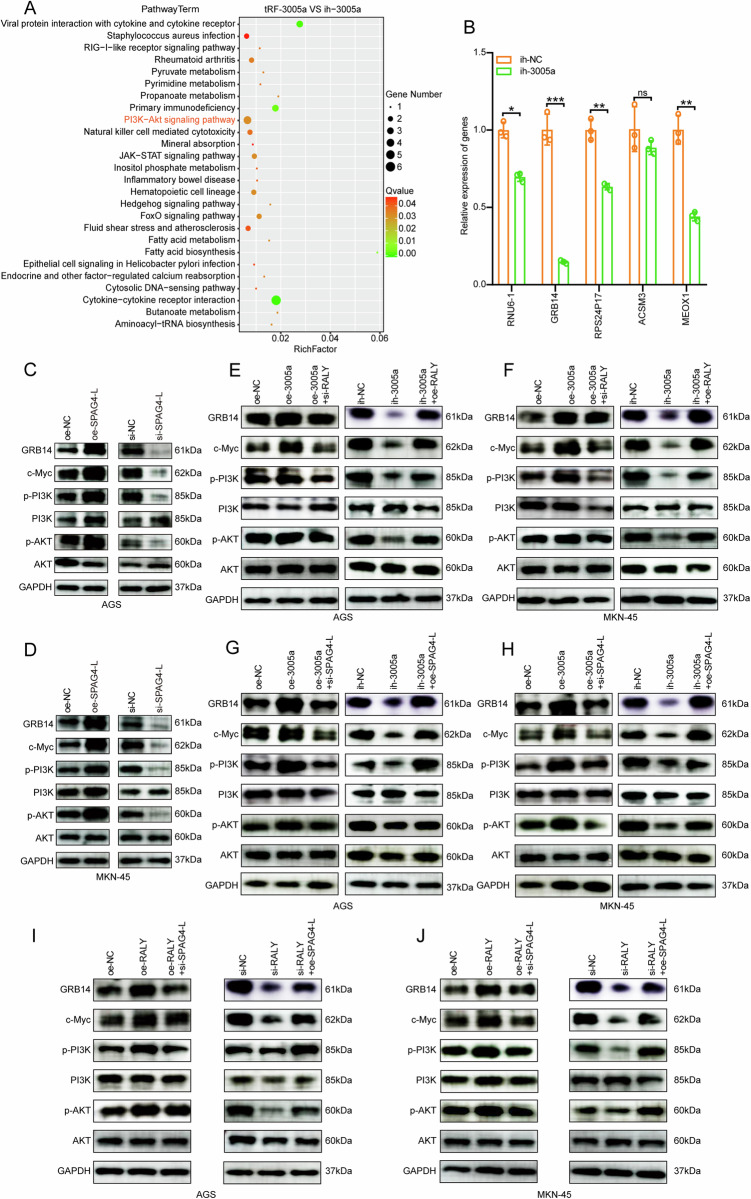
tRF-3005a–RALY axis regulates the GRB14/PI3K/AKT pathway. **A** Bubble plot of KEGG pathway enrichment. Bubble size represents gene count, and color denotes *q*-value. **B** qRT-PCR assays were performed to detect the expression level of downstream differentially expressed candidate genes in MKN-45 cells transfected with ih-NC, ih-3005a. **C**, **D** WB assays were performed to detect the expression level of pathway proteins in AGS and MKN-45 cells transfected with oe-NC or oe-SPAG4-L; si-NC or si-SPAG4-L. **E**, **F** WB assays were performed to detect the expression level of pathway proteins in AGS and MKN-45 cells transfected with oe-NC, oe-3005a, or oe-3005a+si-RALY; ih-NC, ih-3005a, or ih-3005a+oe-RALY. **G**, **H** WB assays were performed to detect the expression level of pathway proteins in AGS and MKN-45 cells transfected with oe-NC, oe-3005a, or oe-3005a+si-SPAG4-L; ih-NC, ih-3005a, or ih-3005a+oe-SPAG4-L. **I**, **J** WB assays were performed to detect the expression level of pathway proteins in AGS and MKN-45 cells transfected with oe-NC, oe-RALY, or oe-RALY+si-SPAG4-L; si-NC, si-RALY, or si-RALY+oe-SPAG4-L. Data were shown as mean ± SD. (Student’s *t*-test, **P < 0.01, ***P < 0.001). NC means no significant.

To explore the downstream mechanisms of the tRF-3005a–RALY axis, sequencing analysis was performed to identify SPAG4-associated pathways and differentially expressed genes. The analysis highlighted the PI3K–AKT pathway as the key downstream signaling cascade (Fig. 6A). Among the differentially expressed genes (Table S6), candidates were filtered based on fold change, and qRT-PCR analysis in tRF-3005a knockdown cells revealed that GRB14 exhibited the most significant decrease in expression (Fig. 6B). Therefore, we speculate that the tRF-3005a–RALY–SPAG4 axis promotes tumor progression through the GRB14/PI3K/AKT pathway. To validate this hypothesis, we first performed loss-of-function experiments of GRB14 and pharmacological blockade of the PI3K/AKT pathway. As shown in the results, GRB14 depletion significantly attenuated the proliferative effects induced by tRF-3005a or SPAG4-L overexpression, as assessed by CCK-8 and colony formation assays (Fig. S5A–D). In parallel, pharmacological inhibition of PI3K/AKT signaling with LY294002 in tRF-3005a or SPAG4-L overexpressing models markedly suppressed cell proliferation in CCK-8 and colony formation assays (Fig. S5E–H). Together, these results suggest that the GRB14/PI3K/AKT pathway may be required for the tRF-3005a–RALY–SPAG4-L axis to promote tumor progression. Moreover, reintroduction of GRB14 into GRB14-depleted cells restored the malignant phenotypes, confirming pathway specificity (Fig. S5A–D).

To further validate our hypothesis, we assessed the protein expression of each gene by Western blotting. The results indicated that overexpression or knockdown of SPAG4-L increased or decreased the protein levels of GRB14, c-Myc, p-PI3K, and p-AKT, respectively (Fig. 6C, D). And other results showed that tRF-3005a altered GRB14, c-Myc, p-PI3K, and p-AKT expression, which was reversed by RALY or SPAG4-L modulation (Fig. 6E–H). Moreover, modulating RALY expression also affected the protein levels of GRB14, c-Myc, p-PI3K, and p-AKT, and this effect could be reversed by regulating SPAG4-L (Fig. 6I, J). Collectively, these results support a model in which the tRF-3005a–RALY axis modulates SPAG4 alternative splicing, thereby regulating GRB14 and activating PI3K/AKT signaling to promote tumor progression.

### Inhibition of tRF-3005a restrains the growth of GC in vivo

**Fig. 7:**
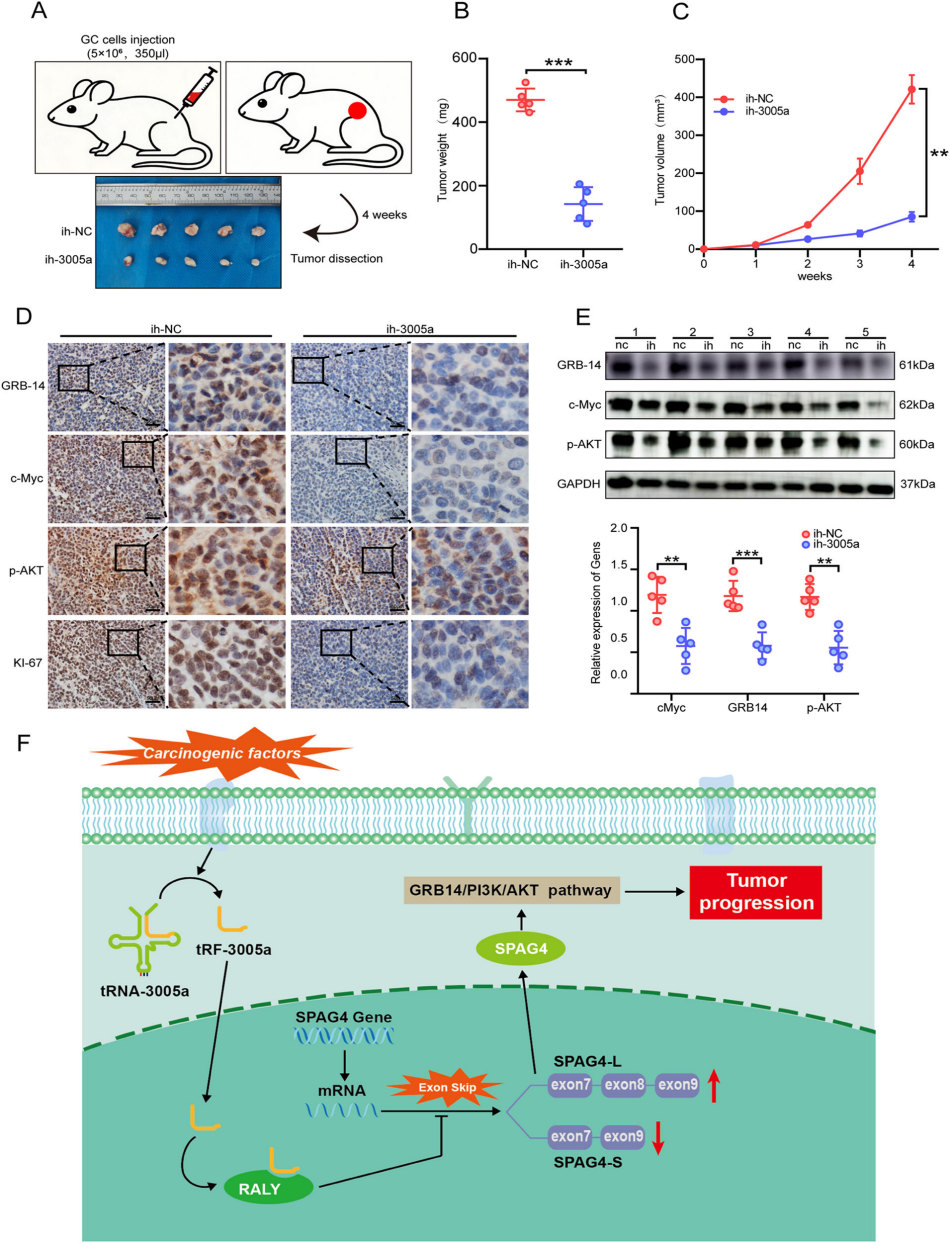
Inhibition of tRF-3005a inhibits the growth of GC in vivo. **A** Schematic workflow of subcutaneous tumorigenesis. **B**, **C** The weights and volumes of tumors were significantly decreased in the ih-3005a group than those in the ih-NC group. **D**, **E** IHC and WB results showed that the expressions of GRB14, c-Myc, p-AKT, and KI-67 were downregulated in the ih-3005a group compared with the ih-NC group. **F** Schematic representation of the mechanism. Through direct binding, tRF-3005a induced conformational changes in RALY and reinforced its repression of SPAG4 exon 8 skipping, thereby activating the GRB14/PI3K/AKT pathway and driving GC progression. Data were shown as mean ± SD. (Student’s *t*-test, **P < 0.01, ***P < 0.001).

To further investigate the role of tRF-3005a in vivo, subcutaneous tumor formation assays were performed in nude mice. Stable ih-3005a or ih-NC transfected MKN-45 cells (5 × 10^6^, 350 μl) were subcutaneously implanted into the lower back of nude mice (5 mice per group). Tumor volumes were assessed weekly, and tumors were dissected after 4 weeks (Fig. 7A). The ih-3005a group showed a significant reduction in tumor weight and volume relative to the ih-NC group (Fig. 7B, C). Additionally, significant downregulation of GRB14, c-Myc, p-AKT, and KI-67 was observed in the tRF-3005a group by IHC assays (Fig. 7D). And WB data were consistent with IHC results (Fig. 7E). Overall, our findings indicate that silencing tRF-3005a inhibits GC growth in vivo.

In summary, tRF-3005a may directly bind to RALY and potentially induce conformational changes, which could enhance RALY-mediated inhibition of SPAG4 exon 8 skipping, thereby activating the GRB14/PI3K/AKT signaling pathway and ultimately promoting GC progression (Fig. 7F).

## Discussion

In this study, we uncovered a novel oncogenic role of the tRF-3005a–RALY regulatory axis in gastric cancer (GC). Through sequencing, molecular biology, and in vivo experimental analyses, we demonstrated that tRF-3005a is highly expressed in GC and correlates with poor prognosis in patients. Mechanistically, tRF-3005a can bind to the RALY protein, may alter its conformation, and enhance RALY-mediated repression of exon 8 skipping in the SPAG4 gene. This, in turn, activates the GRB14/PI3K/AKT signaling pathway, ultimately promoting GC progression.

Our findings support the notion that tRNA-derived fragments (tRFs) are functional small RNAs rather than degradation byproducts [41, 42]. Consistent with previous reports that tRF-Val, tRF-Tyr, tRF-33, tRF-27, and tiRNA-Gly regulate cancer via RNA-binding proteins [15–18, 43], our study reveals that tRF-3005a, which is enriched in the nucleus, can modulate alternative splicing (AS). This uncovers a novel mechanism by which tRFs influence tumor biological functions through the regulation of splicing factors.

RALY, a member of the heterogeneous nuclear ribonucleoprotein (hnRNP) family, exerts diverse roles in tumorigenesis. These include promoting hepatocellular carcinoma cell proliferation by regulating the export of USP22 mRNA [44], suppressing p53 protein activity in lung cancer [45], and reprogramming mitochondrial metabolism via the regulation of miRNA processing in colorectal cancer [46], among others. Furthermore, previous studies have demonstrated its involvement in splicing regulation in cancer [26, 29, 47]. The present study further uncovers the role of RALY in gastric cancer (GC): it promotes the production of the oncogenic SPAG4-L isoform (long isoform) by inhibiting exon 8 skipping of the SPAG4 gene. Notably, the binding of tRF-3005a to RALY enhances its splicing regulatory activity, suggesting a cooperative interaction between small noncoding RNAs (sncRNAs) and splicing factors in tumor progression.

Downstream analyses indicated that the GRB14/PI3K/AKT signaling pathway is a key effector pathway of the tRF-3005a–RALY–SPAG4 regulatory axis. Growth factor receptor-bound protein 14 (GRB14), a modulator of the receptor tyrosine kinase signaling pathway, is crucial for regulating cellular signal transduction and growth. Its role in tumor biology has attracted increasing attention [48, 49]. The PI3K/AKT signaling pathway is indispensable for multiple cellular functions and is frequently dysregulated in cancers, driving tumor initiation and progression [50]. Our experimental data demonstrated that altering the expression levels of tRF-3005a or RALY affects the expression of GRB14 and the activity of the PI3K/AKT pathway, and this effect can be reversed by modulating the alternative splicing of SPAG4. Therefore, tRF-3005a promotes gastric cancer (GC) progression through the tRF-3005a–RALY–SPAG4-GRB14/PI3K/AKT cascade (signaling cascade).

Despite the novel insights gained from this study, it has several limitations. First, the mechanistic link between tRF-3005a and RALY conformational changes remains speculative and requires further validation using high-resolution structural techniques (e.g., cryo-electron microscopy [cryo-EM] or nuclear magnetic resonance [NMR] spectroscopy). Second, although in vivo models have confirmed the oncogenic role of tRF-3005a, the small sample size means its conclusions need to be validated in larger cohorts or patient-derived models. Third, this study focused solely on exon 8 of the SPAG4 gene, leaving the broad impact of the tRF-3005a–RALY regulatory axis on genome-wide alternative splicing events unexplored. Finally, the clinical potential of tRF-3005a as a biomarker or therapeutic target is still in its preliminary stage and requires prospective multicenter studies for further validation.

## Conclusion

Our study reveals that tRF-3005a binds to and modulates the RALY protein, repressing exon 8 skipping of the SPAG4 gene while activating the GRB14/PI3K/AKT signaling pathway, ultimately driving gastric cancer (GC) progression. These findings expand our understanding of the functional repertoire of tRNA-derived fragments (tRFs) and highlight the interplay between small noncoding RNAs (sncRNAs) and splicing factors in cancer. Our research not only uncovers novel regulatory complexity in GC but also identifies potential therapeutic targets for this disease.

## Materials and methods

### Cell lines and cell culture

Human gastric cancer cell lines (AGS, MKN-45, MKN-28, and HGC-27) and the normal gastric epithelial cell line (GES-1) were obtained from the Cell Bank of the Chinese Academy of Sciences (Shanghai, China). Cells were maintained in RPMI-1640 medium (Gibco, USA) supplemented with 10% FBS and 1% penicillin–streptomycin, and cultured at 37 °C in a humidified incubator with 5% CO₂. All cell lines were authenticated by short tandem repeat (STR) analysis and verified to be free of mycoplasma contamination.

### Human tissue specimens

Gastric cancer tissues and matched adjacent normal tissues were collected from patients who underwent surgical resection at Shandong Provincial Hospital between 2020 and 2025, after obtaining informed consent. All patients had a definite preoperative and postoperative pathological diagnosis of gastric adenocarcinoma, and none of them received neoadjuvant therapy prior to surgery. All samples were immediately snap-frozen in liquid nitrogen and stored long-term at –80 °C. The clinical data were collected by experienced physicians. The researchers were blinded to the clinical data during the course of the study. This study was conducted in accordance with the principles of the Declaration of Helsinki and approved by the Ethics Committee of Shandong Provincial Hospital.

### RNA sequencing and data analysis

The quality control of the RNA sequencing library was assessed using the Agilent Bioanalyzer 2100 to ensure that the RNA samples were intact, free of degradation, and of high purity, thereby guaranteeing the accuracy and reliability of the subsequent sequencing results. tRFs and tiRNAs sequencing and data analysis were conducted following our established protocol [15].

### Quantitative real-time PCR (qRT-PCR)

RNA was isolated from human GC tissues and cell lines using TRIzol (Takara, Japan). mRNA reverse transcription was performed in a 10 μl reaction system, following the protocol outlined in previous studies [15, 16]. cDNA amplification was conducted using qRT-PCR with SYBR Green Pro Taq HS Premix (Accurate, Hunan, China) on the Light Cycler 480 system (Roche Diagnostics, Basel, Switzerland). β-actin acted as the internal control for mRNA, with U6 acting as the internal control for tRFs. Expression levels of genes normalized to the internal controls were measured using the 2^−ΔΔCt^ method, and all assays were conducted in triplicate. Primer sequences are listed in Table S1.

### Cell transfection

The tRF-3005a overexpression model (oe-3005a) was established by transfecting tRF-3005a mimics, while the control (oe-NC) was transfected with negative control oligonucleotides. The inhibition model (ih-3005a) was established by transfecting tRF-3005a inhibitors, while the control group (ih-NC) was transfected with negative control inhibitors. The overexpression models of other genes in this study (oe-RALY, oe-SPAG4/oe-SPAG4-L/SPAG4-L, SPAG4-S) were constructed by synthesizing pcDNA vectors, while small interfering RNAs (siRNAs) were synthesized to knock down gene expression (si-RALY, si-SPAG4/si-SPAG4-L). Corresponding controls were also synthesized for both models (oe-NC, si-NC of each model). Lipofectamine 3000(Invitrogen USA) was used for transfection, following the protocol of the manufacturer. All transfection sequences were synthesized based on previous studies [15, 16] and are listed in Table S2.

### CCK-8 proliferation and colony formation assays

For the CCK-8 assay, cells were seeded in 96-well plates 24 h post-transfection and cultured continuously for 4 days. During this period, 10 μl of CCK-8 solution was added to each well at 0, 24, 48, 72, and 96 h, followed by incubation at 37 °C for 2 h. The optical density (OD) at 450 nm for each well was measured using the multifunctional microplate reader (Thermo Fisher Scientific, MA, USA). All assays were conducted in triplicate. For the colony formation assay, transfected cells were seeded into six-well plates at a density of 500–800 cells per well and cultured at 37 °C in a 5% CO₂ incubator for 10–12 days. Cells were stained with crystal violet for 30 min, then used ImageJ software (NIH) to count the colonies. All assays were conducted in triplicate.

### Transwell invasion assay

Invasion assays were conducted using Transwell chambers (Corning, NY, USA) with 8-μm pore polycarbonate membranes that had been precoated with Matrigel (3 mg/ml; BD, NJ, USA). 5 × 10^4^ transfected GC cells suspended in 200 μL serum-free medium were seeded in the upper chambers, and medium with 10% FBS was placed in the lower chambers as a chemoattractant. After incubation for 24 h in a humidified incubator (37 °C, 5% CO₂), the cells remaining on the upper surface of the membrane were removed. Cells on the lower surface were fixed with paraformaldehyde and subsequently stained with crystal violet. Finally, capture images under a microscope (Olympus, Tokyo, Japan) at ×200 magnification and count the cells using ImageJ. All assays were conducted in triplicate.

### Scratch healing assay

Transfected and control cells were seeded into six-well plates, cultured to near confluence, and then scratched with a sterile 200-µL pipette tip to create a wound. After PBS washing to remove detached cells, images were captured under a microscope (Olympus, ×100) at 0 and 24 h of incubation. Wound closure was subsequently quantified using ImageJ software. All assays were conducted in triplicate.

### RNA pull-down assay

Biotin-labeled tRF-3005a, antisense probes were designed and synthesized by BioSune (Shanghai, China). RNA pull-down assays were conducted following established procedures [15]. Silver staining was performed using the Fast Silver Stain Kit (Beyotime, Shanghai, China). Proteins were identified by mass spectrometry at the Advanced Medical Research Institute of Shandong University (Jinan, China). RNA pull-down probes are listed in Table S3.

### RNA immunoprecipitation (RIP)

The RNA Immunoprecipitation (RIP) Kit (Geneseed, Guangzhou, China) was employed according to the protocol of the manufacturer. Initially, GC cells were disrupted in complete RIP lysis buffer. Then, magnetic beads conjugated with anti-RALY (Proteintech, Wuhan, China) or anti-IgG (Proteintech) were incubated with the extract. Finally, RNA bound to proteins was extracted, purified, and quantified using qRT-PCR.

### Western blot (WB) assay

Western blotting was conducted in accordance with established protocols [15, 16]. Antibodies were employed in this study: anti-RALY (Protentech), anti-GRB14 (Protentech), anti-PI3K (Cell Signaling Technology, MA, USA), anti-p-PI3K (CST), anti-AKT (CST), anti-p-AKT (CST), and anti-c-Myc (Protentech). α-tubulin (Protentech) was employed as an internal reference for RALY to ensure equal protein loading. And GAPDH (Protentech) served for other protein normalization. The original Western blot images are provided in the Supplementary Materials.

### Fluorescence in situ hybridization (FISH)

FISH assays were performed according to a previously established protocol [15, 16]. A Cy3-labeled probe targeting tRF-3005a was designed and synthesized by GenePharma (Shanghai, China). U6 and 18S acted as reference controls for nuclear and cytoplasmic RNA. FISH probes are listed in Table S4.

### Immunohistochemistry (IHC)

The IHC staining assay was conducted using the IHC Kit (Zsgb, Bio, Beijing, China) following the manufacturer’s protocol, and performed on 5-μm-thick paraffin-embedded tissue sections. After deparaffinization, hydration, and antigen retrieval, endogenous peroxidase activity was blocked, and sections were incubated with goat serum. Primary antibodies were applied at 2–8 °C overnight, followed by HRP-conjugated secondary antibodies at 37 °C for 30 min. Slides were then developed with DAB, counterstained with hematoxylin, and imaged under a microscope (Olympus).

### Tumorigenesis assay in vivo

To assess the in vivo effect of tRF-3005a on tumor progression, 4-week-old BALB/c nude mice were used for tumorigenesis experiments. Nude mice were purchased from Charles River Laboratory (Beijing, China) and kept at the Experimental Animal Center of Shandong Provincial Hospital. MKN-45 cells that stably silenced tRF-3005a (ih-3005a) and control cells (ih-NC) were subcutaneously injected into the nude mice, which were divided randomly into groups, with 5 animals per group. Tumor sizes were recorded weekly, and after 4 weeks, the mice were sacrificed for tumor collection. The excised tumors were weighed and subjected to WB and IHC analyses. All procedures were conducted with approval from the Animal Ethics Committee of Shandong Provincial Hospital.

### Statistical analysis

Statistical analyses were performed using SPSS 26.0 (IBM, Chicago, USA). Differences between the two groups were assessed by Student’s *t*-test or Mann–Whitney *U*-test, and a paired *t*-test was used for comparisons in 72 paired tumor and adjacent normal tissues. Associations between tRF-3005a expression and clinicopathological features were analyzed by the χ^2^ test. Overall survival (OS) was evaluated using the log-rank test. Data are presented as mean ± SD. A *P*-value < 0.05 was considered statistically significant (*P < 0.05, **P < 0.01, ***P < 0.001).

## Supplementary information

Original Western blots

Figure S1

Figure S2

Figure S3

Figure S4

Figure S5

Supplementary figure legends

Supplementary Table 1

Supplementary Table 2

Supplementary Table 3

Supplementary Table 4

Supplementary Table 5

Supplementary Table 6

## Data Availability

The datasets used and/or analyzed during the current study are available from the corresponding author on reasonable request.
