# Supplementary figures and images for: tRF-3005a regulates exon skipping of SPAG4 by interacting with RALY to drive gastric cancer progression
Source: Cell Death Discov. 2026 Mar 24;12:169. doi: 10.1038/s41420-026-03049-3 (PMC13039196; doi:10.1038/s41420-026-03049-3)

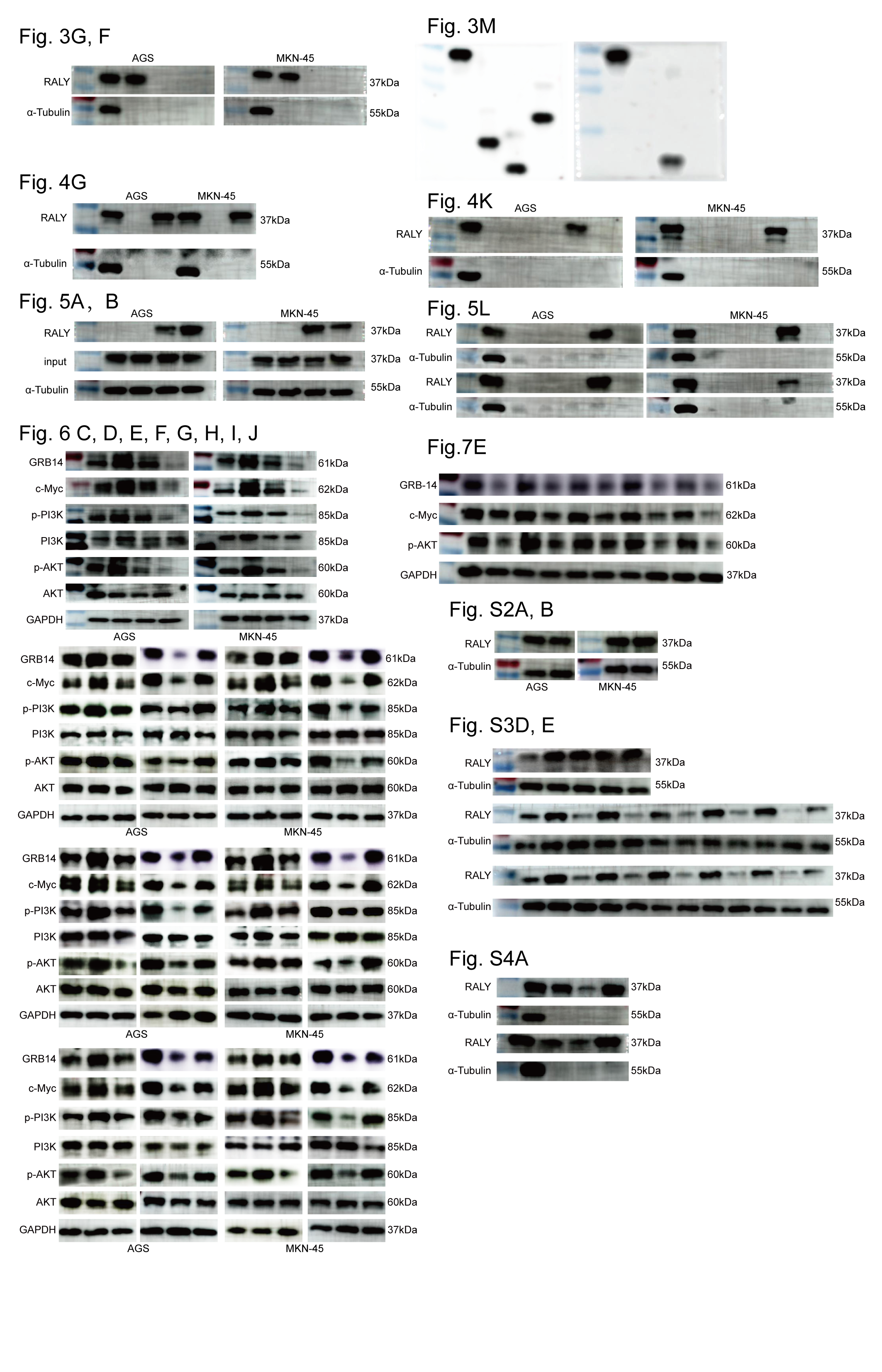

Supplement: Supplementary file 1 — Original Western blots [file 41420_2026_3049_MOESM1_ESM.tif]

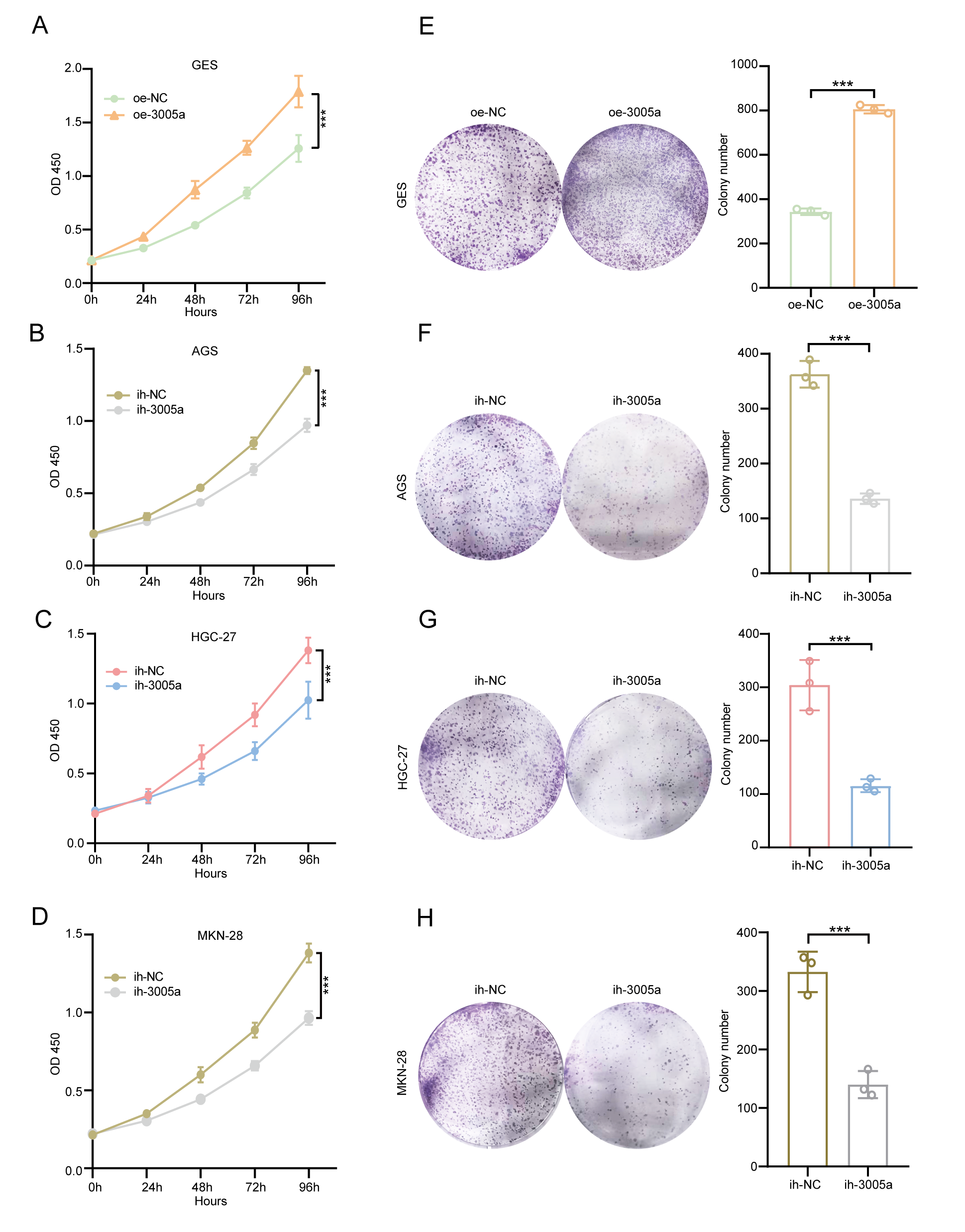

Supplement: Supplementary file 2 — Figure S1 [file 41420_2026_3049_MOESM2_ESM.tif]

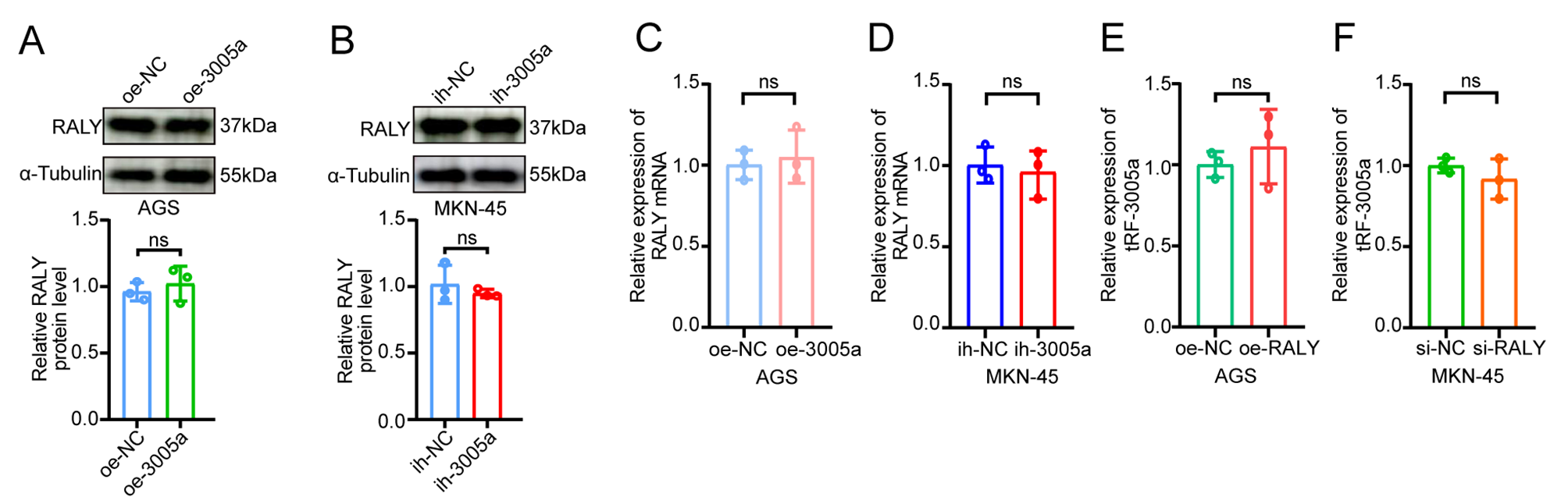

Supplement: Supplementary file 3 — Figure S2 [file 41420_2026_3049_MOESM3_ESM.tif]

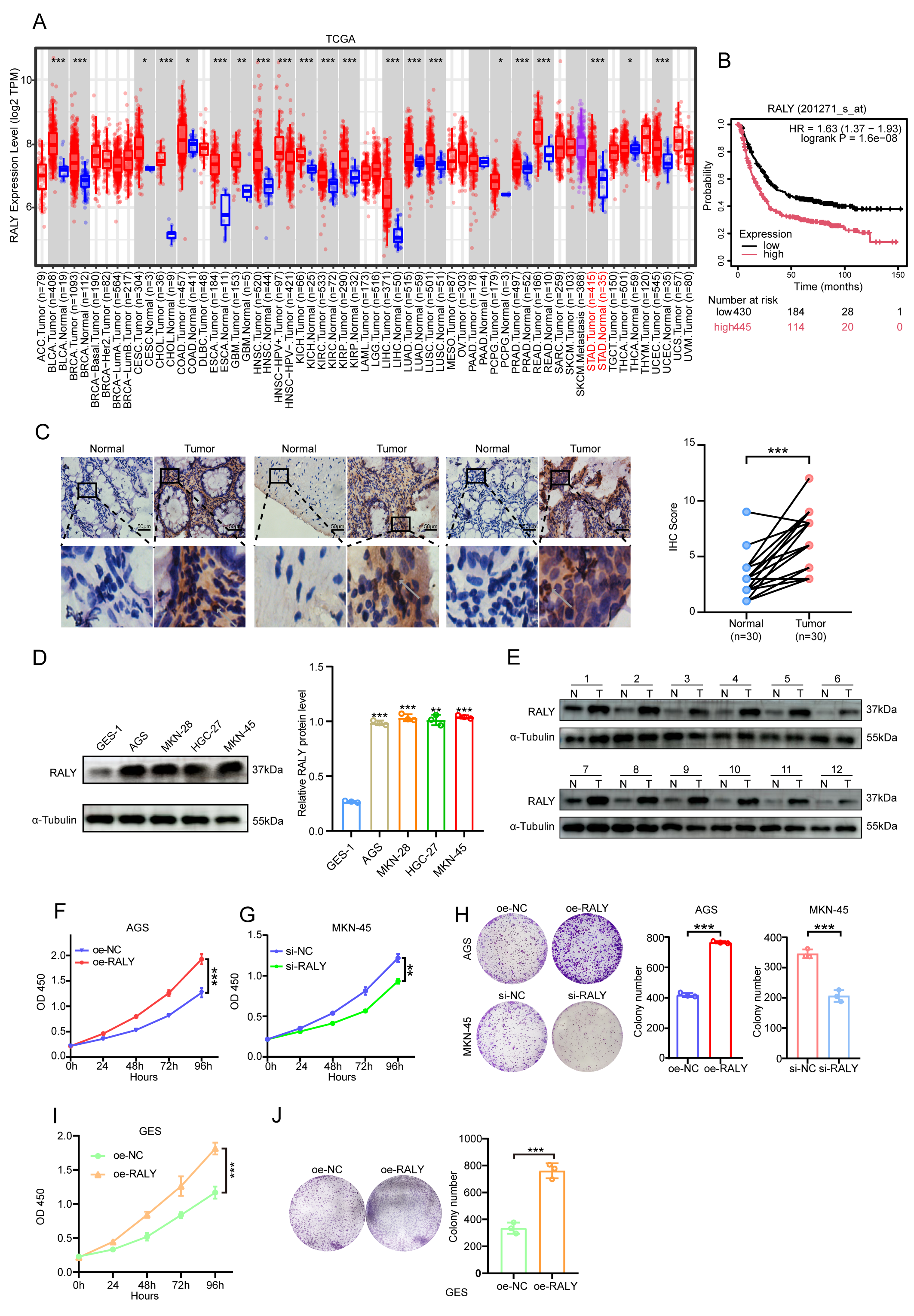

Supplement: Supplementary file 4 — Figure S3 [file 41420_2026_3049_MOESM4_ESM.tif]

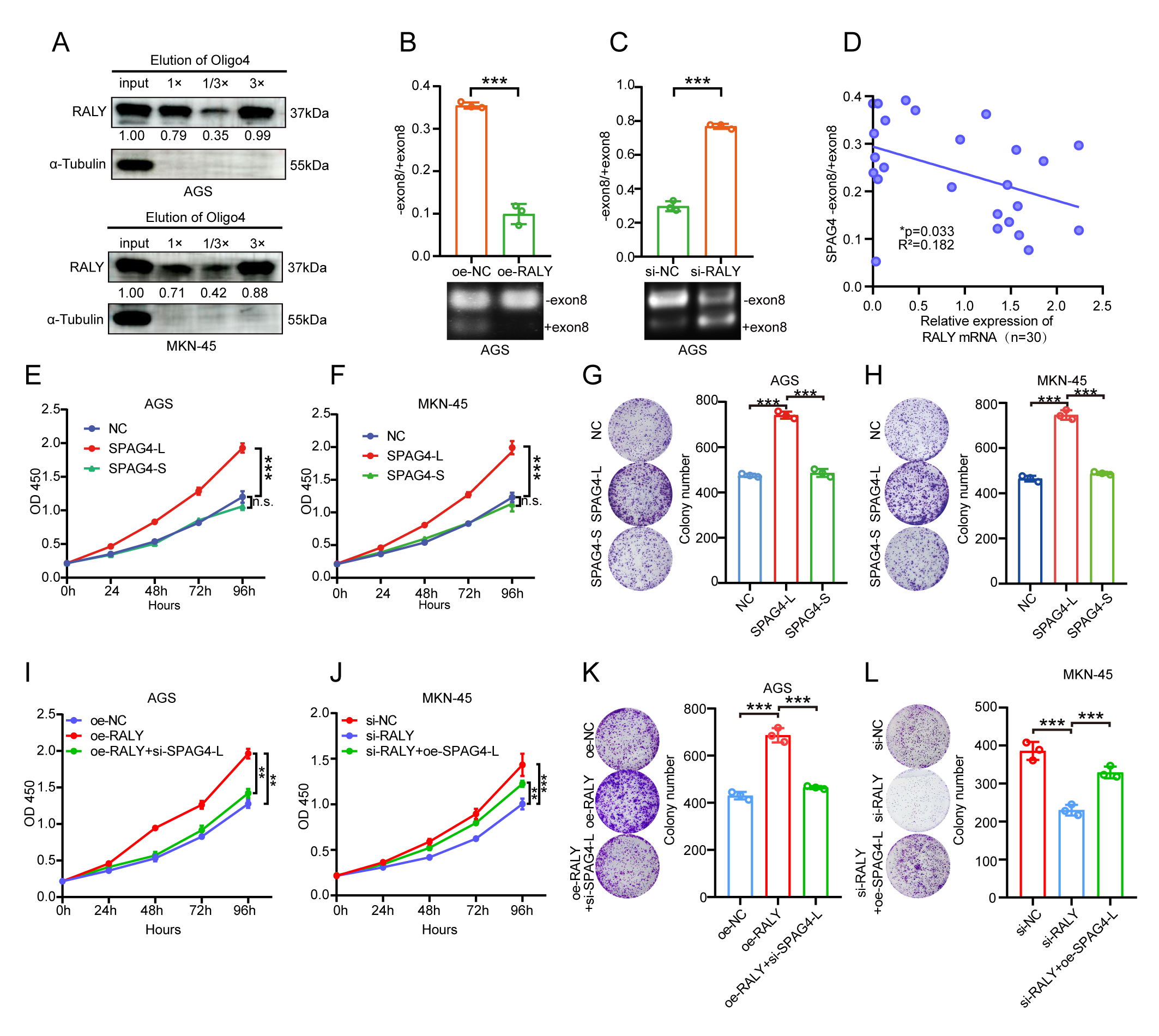

Supplement: Supplementary file 5 — Figure S4 [file 41420_2026_3049_MOESM5_ESM.tif]

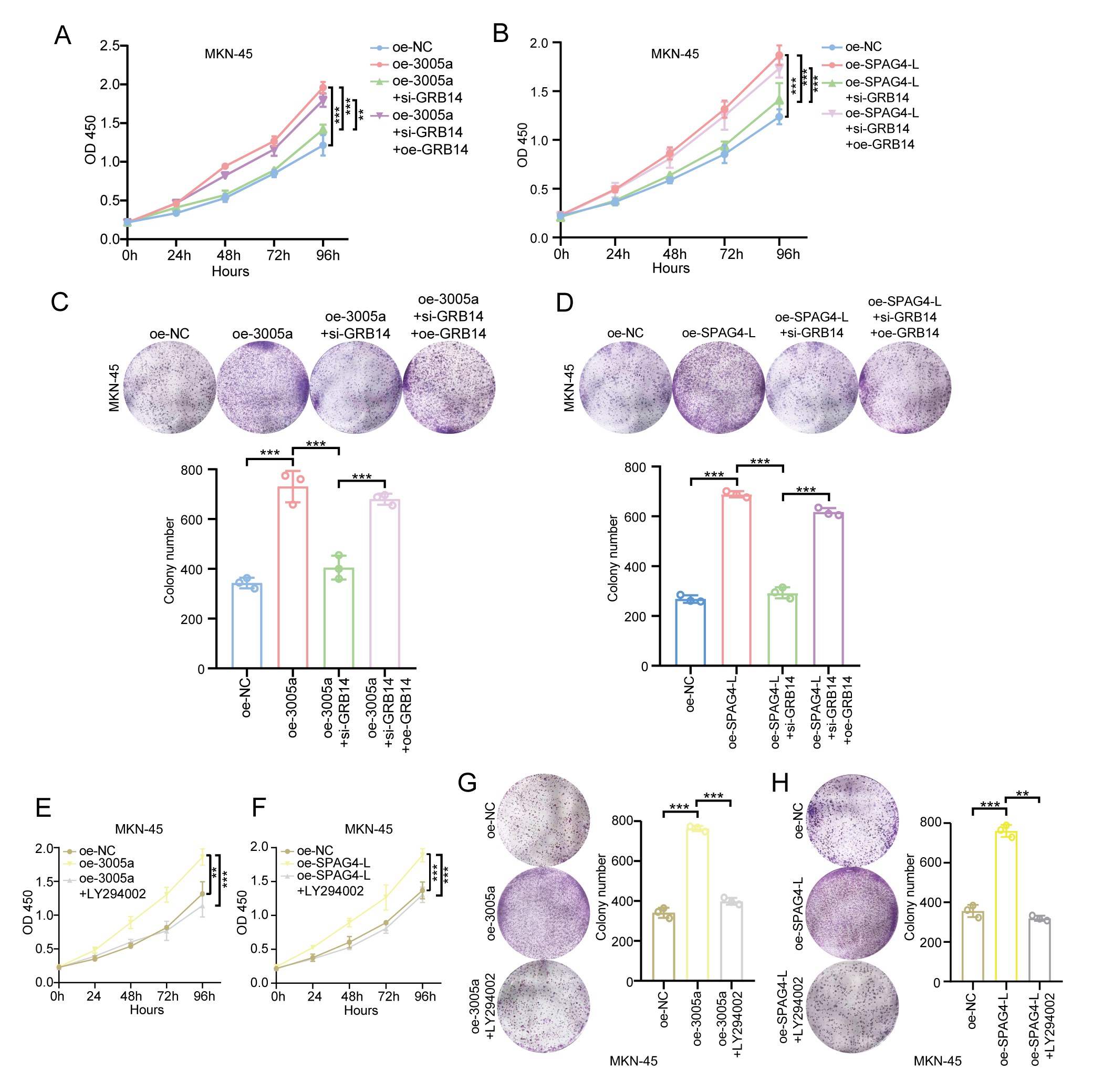

Supplement: Supplementary file 6 — Figure S5 [file 41420_2026_3049_MOESM6_ESM.tif]
