# Supplementary material for: tRF-3005a regulates exon skipping of SPAG4 by interacting with RALY to drive gastric cancer progression
Source: Cell Death Discov. 2026 Mar 24;12:169. doi: 10.1038/s41420-026-03049-3 (PMC13039196; doi:10.1038/s41420-026-03049-3)
Supplement: Supplementary file 7 — Supplementary figure legends [file 41420_2026_3049_MOESM7_ESM.docx]

Supplementary information

Additional file 1: **Figure S1 related to Figure 2. tRF-3005a promotes proliferation of GES-1 cells.** **A, B, C, D** CCK-8 assays were performed to detect the proliferation of GES-1 cells transfected with oe-NC or oe-3005a **(A),** and AGS, HGC27 and MKN-28 cells transfected with ih-NC or ih-3005a **(B, C, D)**. **E, F, G, H** Colony formation assays were performed to detect the proliferation of GES-1 cells transfected with oe-NC or oe-3005a **(E)**, and AGS, HGC27 and MKN-28 cells transfected with ih-NC or ih-3005a **(F, G, H)**.

Additional file 2: **Figure S2 related to Figure 3. Interaction between tRF-3005a and RALY. A, B** WB assays were conducted to identify the expression level of RALY protein in AGS cells transfected with oe-NC or oe-3005a, and MKN-45 cells transfected with ih-NC or ih-3005a**.** **C, D** qRT-PCR assays were conducted to identify the expression level of RALY gene in AGS cells transfected with oe-NC or oe-3005a, and MKN-45 cells transfected with ih-NC or ih-3005a**.** **E, F** qRT-PCR assays were conducted to identify the expression level of tRF-3005a in AGS cells transfected with oe-NC or oe-RALY, and MKN-45 cells transfected with si-NC or si-RALY**.** Data were expressed as mean±SD. (Student′s t test; *, P<0.05; **, P<0.01, and ***, P<0.001). ns means no significant.

Additional file 3: **Figure S3 RALY is upregulated in GC and promoted proliferation in vitro. A** The expression levels of the RALY gene in different tumors from TCGA by Timer. **B** Overall survival analysis of the RALY in GC by Kaplan-Meier Plotter. **C** IHC assays were conducted to identify the expression level of RALY in GC tissues and adjacent normal tissues. **D, E** WB assays were conducted to identify the expression level of RALY protein in GES-1 cells, GC cell lines, normal and GC tissues. **F, G, I** CCK-8 assays were performed to detect the proliferation of AGS and GES-1 cells transfected with oe-NC or oe-RALY (**F, I**), and MKN-45 cells transfected with si-NC or si-RALY (**G**). **H** Colony formation assays were performed to detect the proliferation of AGS and GES-1 cells transfected with oe-NC or oe-RALY and MKN-45 cells transfected with si-NC or si-RALY. **J** Colony formation assays were performed to detect the proliferation of GES-1 cells transfected with oe-NC or oe-RALY. Data were expressed as mean±SD. (Student′s t test; *, P<0.05; **, P<0.01, and ***, P<0.001).

Additional file 4: **Figure S4 related to Figure 4. RALY promotes GC Cells proliferation by inhibiting SPAG4 exon skipping. A** Oligo4 RNA pull-down assays were performed to detect the specific interaction site between RALY and SPAG4. **B, C** qRT-PCR assays were conducted to show the ratio of SPAG4-S/SPAG4-L in AGS cells transfected with oe-NC, oe-RALY, si-NC or si-RALY. **D** Correlation analysis of RALY expression level and the ratio of SPAG4-S/L in 30 GC tissues by qRT-PCR. **E, F** CCK-8 assays were performed to detect the proliferation of AGS and MKN-45 cells transfected with NC, SPAG4-L or SPAG4-S. **G, H** Colony formation assays were performed to detect the proliferation of AGS and MKN-45 cells transfected with NC, SPAG4-L or SPAG4-S. **I, J** CCK-8 rescue assays were performed to detect the proliferation of AGS cells transfected with oe-NC, oe-RALY or oe-RALY+si-SPAG4-L, and MKN-45 cells transfected with ih-NC, ih-RALY or ih-RALY+oe-SPAG4-L. **K, L** Colony formation rescue assays were performed to detect the proliferation of AGS cells transfected with oe-NC, oe-RALY or oe-RALY+si-SPAG4-L, and MKN-45 cells transfected with ih-NC, ih-RALY or ih-RALY+oe-SPAG4-L. Data were expressed as mean±SD. (Student′s t test, Pearson correlation test with two-tailed; *, P<0.05; **, P<0.01, and ***, P<0.001).

Additional file 5: **Figure S5 related to Figure 6. tRF-3005a–RALY-SPAG4 axis regulates the** **GRB14/PI3K/AKT pathway. A, B** CCK-8 assays were performed to detect the proliferation of MKN-45 cells transfected with oe-NC, oe-3005a, oe-3005a+si-GRB14 or oe-3005a+si-GRB14+oe-GRB14 **(A)**, and oe-NC, oe-SPAG4-L, oe-SPAG4-L+si-GRB14 or oe-SPAG4-L+si-GRB14+oe-GRB14 **(B)**. **C, D** Colony formation assays were performed to detect the proliferation of MKN-45 cells transfected with oe-NC, oe-3005a, oe-3005a+si-GRB14 or oe-3005a+si-GRB14+oe-GRB14 **(C)**, and oe-NC, oe-SPAG4-L, oe-SPAG4-L+si-GRB14 or oe-SPAG4-L+si-GRB14+oe-GRB14 **(D)**. **E, F, G, H** First, tRF-3005a or SPAG4-L was overexpressed in MKN-45 cells, followed by treatment with the PI3K inhibitor LY294002. Subsequently, CCK-8 **(E, F)** and colony formation assays were performed to assess cell proliferation **(G, H)**. (Student′s t test; *, P<0.05; **, P<0.01, and ***, P<0.001).
