# Supplementary material for: tRF-3005a regulates exon skipping of SPAG4 by interacting with RALY to drive gastric cancer progression
Source: Cell Death Discov. 2026 Mar 24;12:169. doi: 10.1038/s41420-026-03049-3 (PMC13039196; doi:10.1038/s41420-026-03049-3)
Supplement: Supplementary file 8 — Supplementary Table 1 [file 41420_2026_3049_MOESM8_ESM.docx]

Supplementary Table 1 The primer sequences of the genes in this experiment.

| Genes | Primer sequences (5’ to 3’) |
| --- | --- |
| tRF-3005a | ATCTCGGTGGGACCTCCAAA |
| U6 | F: GGAACGATACAGAGAAGATTAGC |
|  | R: TGGAACGCTTCACGAATTTGCG |
| RALY | F: AGGGCTATGCCTTTGTTCAGT |
|  | R: CCTTGGGTCTGTCAGGCTTAG |
| SPAG4/SPAG4-L | F: AAGGAGGTGTCCACTGTTCG |
|  | R: CTGTCTGCGTAATCGTGGGA |
| SPAG4-S | F: AACAGCGAGGAGCCTCCAT |
|  | R: CCAGAAGCTGAAGCGATTCC |
| SPAG4-exon2 | F: ACCGACTGGGTCTCCAGTAGT |
|  | R: CAGAAAGCTCTTGAACACCCG |
| SPAG4-exon8 | F: GCCAAGCTCGTGTTCCAGAG |
|  | R: GCTCAAAGCATAGTCGGGCT |
| SPAG4-exon11 | F: GGCCTCCAGGTTTATGATGAA |
|  | R: GCAGGTGGAAAGTCTGAATCTC |
| LUC7L2 | F: CGTAGCCACCAGAGAAGTCG |
|  | R: TAGCACTCTCATAGGACCGC |
| MYEOV | F: GACAGGGACTCGTTGCTCAT |
|  | R: GCACCCTTGTCTCCCTTGTT |
| ZFAND2A-DT | F: TTTGCAGACGCCCATCCTTAT |
|  | R: AGACACCAATACCACCAGGG |
| BLVRB | F: CAATGACCTCAGTCCCACGA |
|  | R: GTGGTCATCAGTCACAGCCT |
| GRB14 | F: AAGGAACATCAAAGGAACCGC |
|  | R: GCCTGCCAGTGACACATAAAT |
| RPS24P17 | F: TTTTCCTCCCTGGATGTCTGAG |
|  | R: CCAGGGTGAAGGACATAGACG |
| RNU6-1 | F: CAGCACATATACTAAAATTGGAACG |
|  | R: ACGAATTTGCGTGTCATCC |
| ACSM3 | F: GGACAGACTGAAACGGTGCT |
|  | R: GGTTTTCCCATTGAGCCAGG |
| MENX1 | F: ACATCTTCACTGAGCAGCACC |
|  | R: TTCTCCGCCTGGATGATTTCT |
| β-action | F: CATGTACGTTGCTATCCAGGC |
|  | R: CTCCTTAATGTCACGCACGAT |
