# Supplementary material for: tRF-3005a regulates exon skipping of SPAG4 by interacting with RALY to drive gastric cancer progression
Source: Cell Death Discov. 2026 Mar 24;12:169. doi: 10.1038/s41420-026-03049-3 (PMC13039196; doi:10.1038/s41420-026-03049-3)
Supplement: Supplementary file 9 — Supplementary Table 2 [file 41420_2026_3049_MOESM9_ESM.docx]

Supplementary Table 2 Oligonucleotide sequences of tRF-3005a used in the cell transfection.

| Name | Oligonucleotide sequences (5’-3’) |
| --- | --- |
| tRF-3005a mimics | UCUCGGUGGGACCUCCA  UGGAGGUCCCACCGAGAUU |
| NC mimics | UUCUCCGAACGUGUCACGUUU  ACGUGACACGUUCGGAGAAUU |
| tRF-3005a inhibitor | UGGAGGUCCCACCGAGA |
| NC inhibitor | ACGUGACACGUUCGGAGAAUU |

| **Sequence for overexpression of RALY in pcDNA3.1 (5’-3’).** |
| --- |
| ATGTCCTTGAAGCTTCAGGCAAGCAATGTAACCAACAAGAATGACCCCAAGTCCATCAACTCTCGAGTCTTCATTGGAAACCTCAACACAGCTCTGGTGAAGAAATCAGATGTGGAGACCATCTTCTCTAAGTATGGCCGTGTGGCCGGCTGTTCTGTGCACAAGGGCTATGCCTTTGTTCAGTACTCCAATGAGCGCCATGCCCGGGCAGCTGTGCTGGGAGAGAATGGGCGGGTGCTGGCCGGGCAGACCCTGGACATCAACATGGCTGGAGAGCCTAAGCCTGACAGACCCAAGGGGCTAAAGAGAGCAGCATCTGCCATATACAGTGGCTACATCTTTGACTATGATTACTACCGGGACGACTTCTACGACAGGCTCTTCGACTACCGGGGCCGTCTGTCGCCCGTGCCAGTGCCCAGGGCGGTCCCTGTGAAGCGACCCCGGGTCACAGTCCCTTTGGTCCGGCGTGTCAAAACTAACGTACCTGTCAAGCTCTTTGCCCGCTCCACAGCTGTCACCACCAGCTCAGCCAAGATCAAGTTAAAGAGCAGTGAGCTGCAGGCCATCAAGACGGAGCTGACACAGATCAAGTCCAATATCGATGCCCTGCTGAGCCGCTTGGAGCAGATCGCTGCGGAGCAAAAGGCCAATCCAGATGGCAAGAAGAAGGGTGATGGAGGTGGCGCCGGCGGCGGCGGCGGTGGTGGTGGCAGCGGTGGCGGTGGCAGTGGTGGTGGCGGTGGCGGTGGCAGCAGCCGGCCACCAGCCCCCCAAGAGAACACAACTTCTGAGGCAGGCCTGCCCCAGGGGGAAGCACGGACCCGAGACGACGGCGATGAGGAAGGGCTCCTGACACACAGCGAGGAAGAGCTGGAACACAGCCAGGACACAGACGCGGATGATGGGGCCTTGCAG |

| **Sequences of the small interfering RNA anti-RALY (5’-3’).** | |
| --- | --- |
| **si-RALY** | **sense**: GAACACAACUUCUGAGGCAUU  **antisense**: UGCCUCAGAAGUUGUGUUCUU |
| **si-NC** | **sense**: UUCUCCGAACGUGUCACGUUU  **antisense**: ACGUGACACGUUCGGAGAAUU |

| **Sequences of the HA-tagged RALY truncated mutants in pcDNA3.1 (5’-3’).** |
| --- |
| **1-92aa:**  ATGTCCTTGAAGCTTCAGGCAAGCAATGTAACCAACAAGAATGACCCCAAGTCCATCGACAGACCCAAGGGGCTAAAGAGAGCAGCATCTGCCATATACAGTGGCTACATCTTTGACTATGATTACTACCGGGACGACTTCTACGACAGGCTCTTCGACTACCGGGGCCGTCTGTCGCCCGTGCCAGTGCCCAGGGCGGTCCCTGTGAAGCGACCCCGGGTCACAGTCCCTTTGGTCCGGCGTGTCAAAACTAACGTACCTGTCAAGCTCTTTGCCCGCTCCACAGCTGTCACCACCAGCTCAGCCAAGATCAAGTTAAAGAGCAGTGAGCTGCAGGCCATCAAGACGGAGCTGACACAGATCAAGTCCAATATCGATGCCCTGCTGAGCCGCTTGGAGCAGATCGCTGCGGAGCAAAAGGCCAATCCAGATGGCAAGAAGAAGGGTGATGGAGGTGGCGCCGGCGGCGGCGGCGGTGGTGGTGGCAGCGGTGGCGGTGGCAGTGGTGGTGGCGGTGGCGGTGGCAGCAGCCGGCCACCAGCCCCCCAAGAGAACACAACTTCTGAGGCAGGCCTGCCCCAGGGGGAAGCACGGACCCGAGACGACGGCGATGAGGAAGGGCTCCTGACACACAGCGAGGAAGAGCTGGAACACAGCCAGGACACAGACGCGGATGATGGGGCCTTGCAG |
| **93-182aa:**  ATGTCCTTGAAGCTTCAGGCAAGCAATGTAACCAACAAGAATGACCCCAAGTCCATCAACTCTCGAGTCTTCATTGGAAACCTCAACACAGCTCTGGTGAAGAAATCAGATGTGGAGACCATCTTCTCTAAGTATGGCCGTGTGGCCGGCTGTTCTGTGCACAAGGGCTATGCCTTTGTTCAGTACTCCAATGAGCGCCATGCCCGGGCAGCTGTGCTGGGAGAGAATGGGCGGGTGCTGGCCGGGCAGACCCTGGACATCAACATGGCTGGAGAGCCTAAGCCTAAGAGCAGTGAGCTGCAGGCCATCAAGACGGAGCTGACACAGATCAAGTCCAATATCGATGCCCTGCTGAGCCGCTTGGAGCAGATCGCTGCGGAGCAAAAGGCCAATCCAGATGGCAAGAAGAAGGGTGATGGAGGTGGCGCCGGCGGCGGCGGCGGTGGTGGTGGCAGCGGTGGCGGTGGCAGTGGTGGTGGCGGTGGCGGTGGCAGCAGCCGGCCACCAGCCCCCCAAGAGAACACAACTTCTGAGGCAGGCCTGCCCCAGGGGGAAGCACGGACCCGAGACGACGGCGATGAGGAAGGGCTCCTGACACACAGCGAGGAAGAGCTGGAACACAGCCAGGACACAGACGCGGATGATGGGGCCTTGCAG |
| **183-306aa:**  ATGTCCTTGAAGCTTCAGGCAAGCAATGTAACCAACAAGAATGACCCCAAGTCCATCAACTCTCGAGTCTTCATTGGAAACCTCAACACAGCTCTGGTGAAGAAATCAGATGTGGAGACCATCTTCTCTAAGTATGGCCGTGTGGCCGGCTGTTCTGTGCACAAGGGCTATGCCTTTGTTCAGTACTCCAATGAGCGCCATGCCCGGGCAGCTGTGCTGGGAGAGAATGGGCGGGTGCTGGCCGGGCAGACCCTGGACATCAACATGGCTGGAGAGCCTAAGCCTGACAGACCCAAGGGGCTAAAGAGAGCAGCATCTGCCATATACAGTGGCTACATCTTTGACTATGATTACTACCGGGACGACTTCTACGACAGGCTCTTCGACTACCGGGGCCGTCTGTCGCCCGTGCCAGTGCCCAGGGCGGTCCCTGTGAAGCGACCCCGGGTCACAGTCCCTTTGGTCCGGCGTGTCAAAACTAACGTACCTGTCAAGCTCTTTGCCCGCTCCACAGCTGTCACCACCAGCTCAGCCAAGATCAAGTTA |

| **Sequence for overexpression of SPAG4/SPAG4-L in pcDNA3.1 (5’-3’).** |
| --- |
| GTGACGTCAGCAGCCGGCCGGGACACAGCGGGAGGGCAGGTGCGGCCGCGGGGCCTGCCGACTTCACGCAGGGTCCGTGGGGTCCCCGCGGCGCGCAGCGGCTGAAGGAGGCCCCAGGGCCTTGGCGACCGCAGCGGCGGCTTTAGCGTCAGTGACTAGGCAGCAGGGGGTCAGGATGCGGCGAAGCTCCCGCCCGGGCTCGGCCTCGTCCTCGCGCAAGCACACGCCCAACTTTTTCAGCGAGAACAGCTCAATGAGCATCACCTCGGAGGACAGCAAAGGGCTCCGGTCAGCGGAGCCCGGGCCTGGGGAGCCCGAGGGCAGAAGAGCCCGGGGCCCGAGCTGCGGTGAGCCCGCCTTGAGCGCGGGAGTGCCCGGAGGAACCACATGGGCAGGAAGCTCTCAGCAGAAGCCAGCGCCTCGGAGCCACAACTGGCAGACAGCCTGTGGCGCGGCAACCGTGAGGGGCGGGGCCTCGGAACCGACTGGGTCTCCAGTAGTCTCTGAGGAGCCGCTCGACCTTCTCCCGACCCTGGATCTGAGGCAGGAGATGCCTCCCCCGCGGGTGTTCAAGAGCTTTCTGAGCCTGCTCTTCCAGGGGCTGAGCGTGTTGTTATCCCTGGCAGGAGACGTGCTGGTCAGCATGTACAGGGAGGTCTGTTCCATCCGCTTCCTGTTCACGGCTGTGTCGCTGCTGAGCCTCTTTCTGTCAGCATTCTGGCTGGGGCTTCTGTACCTGGTCTCTCCTTTGGAGAATGAACCTAAGGAGATGCTGACTCTAAGTGAGTACCACGAGCGCGTGCGCTCCCAGGGGCAGCAGCTGCAGCAGCTCCAGGCCGAGCTGGATAAACTCCACAAGGAGGTGTCCACTGTTCGGGCAGCCAACAGCGAGAGAGTGGCCAAGCTCGTGTTCCAGAGGCTGAATGAGGATTTTGTGCGGAAGCCCGACTATGCTTTGAGCTCTGTGGGAGCCTCCATCGACCTGCAGAAGACATCCCACGATTACGCAGACAGGAACACTGCCTACTTCTGGAATCGCTTCAGCTTCTGGAACTACGCACGGCCGCCCACGGTTATCCTGGAGCCCCACGTGTTCCCTGGGAATTGCTGGGCTTTTGAAGGCGACCAAGGCCAGGTGGTGATCCAACTGCCGGGCCGAGTGCAGCTGAGCGACATCACTCTGCAGCATCCACCGCCCAGCGTGGAGCACACCGGAGGAGCCAACAGCGCCCCCCGCGATTTCGCGGTCTTTGGCCTCCAGGTTTATGATGAAACTGAAGTTTCCTTGGGGAAATTCACCTTCGATGTTGAGAAATCGGAGATTCAGACTTTCCACCTGCAGAATGACCCCCCAGCTGCCTTTCCCAAGGTGAAGATCCAGATTCTAAGCAACTGGGGCCACCCCCGTTTCACGTGCTTGTATCGAGTCCGTGCCCACGGTGTGCGAACCTCAGAGGGGGCAGAGGGCAGTGCACAGGGGCCCCATTAAACATGCTGATTTTTGGAGTAGAATTGAGTTCTGCTGAAGGATACTGGATCAGTGCTTTCGGGGGCTCTGTTG |

| **Sequences of the small interfering RNA anti-SPAG4-L (5’-3’).** | |
| --- | --- |
| **si-SPAG4-L** | **sense**: GUGAAGAUCCAGAUUCUAAUU  **antisense**: UUAGAAUCUGGAUCUUCACUU |
| **si-NC** | **sense**: UUCUCCGAACGUGUCACGUUU  **antisense**: ACGUGACACGUUCGGAGAAUU |

| **Sequence for overexpression of SPAG4-S in pcDNA3.1 (5’-3’).** |
| --- |
| GTGACGTCAGCAGCCGGCCGGGACACAGCGGGAGGGCAGGTGCGGCCGCGGGGCCTGCCGACTTCACGCAGGGTCCGTGGGGTCCCCGCGGCGCGCAGCGGCTGAAGGAGGCCCCAGGGCCTTGGCGACCGCAGCGGCGGCTTTAGCGTCAGTGACTAGGCAGCAGGGGGTCAGGATGCGGCGAAGCTCCCGCCCGGGCTCGGCCTCGTCCTCGCGCAAGCACACGCCCAACTTTTTCAGCGAGAACAGCTCAATGAGCATCACCTCGGAGGACAGCAAAGGGCTCCGGTCAGCGGAGCCCGGGCCTGGGGAGCCCGAGGGCAGAAGAGCCCGGGGCCCGAGCTGCGGTGAGCCCGCCTTGAGCGCGGGAGTGCCCGGAGGAACCACATGGGCAGGAAGCTCTCAGCAGAAGCCAGCGCCTCGGAGCCACAACTGGCAGACAGCCTGTGGCGCGGCAACCGTGAGGGGCGGGGCCTCGGAACCGACTGGGTCTCCAGTAGTCTCTGAGGAGCCGCTCGACCTTCTCCCGACCCTGGATCTGAGGCAGGAGATGCCTCCCCCGCGGGTGTTCAAGAGCTTTCTGAGCCTGCTCTTCCAGGGGCTGAGCGTGTTGTTATCCCTGGCAGGAGACGTGCTGGTCAGCATGTACAGGGAGGTCTGTTCCATCCGCTTCCTGTTCACGGCTGTGTCGCTGCTGAGCCTCTTTCTGTCAGCATTCTGGCTGGGGCTTCTGTACCTGGTCTCTCCTTTGGAGAATGAACCTAAGGAGATGCTGACTCTAAGTGAGTACCACGAGCGCGTGCGCTCCCAGGGGCAGCAGCTGCAGCAGCTCCAGGCCGAGCTGGATAAACTCCACAAGGAGGTGTCCACTGTTCGGGCAGCCAACAGCGAGGAGCCTCCATCGACCTGCAGAAGACATCCCACGATTACGCAGACAGGAACACTGCCTACTTCTGGAATCGCTTCAGCTTCTGGAACTACGCACGGCCGCCCACGGTTATCCTGGAGCCCCACGTGTTCCCTGGGAATTGCTGGGCTTTTGAAGGCGACCAAGGCCAGGTGGTGATCCAACTGCCGGGCCGAGTGCAGCTGAGCGACATCACTCTGCAGCATCCACCGCCCAGCGTGGAGCACACCGGAGGAGCCAACAGCGCCCCCCGCGATTTCGCGGTCTTTGGCCTCCAGGTTTATGATGAAACTGAAGTTTCCTTGGGGAAATTCACCTTCGATGTTGAGAAATCGGAGATTCAGACTTTCCACCTGCAGAATGACCCCCCAGCTGCCTTTCCCAAGGTGAAGATCCAGATTCTAAGCAACTGGGGCCACCCCCGTTTCACGTGCTTGTATCGAGTCCGTGCCCACGGTGTGCGAACCTCAGAGGGGGCAGAGGGCAGTGCACAGGGGCCCCATTAAACATGCTGATTTTTGGAGTAGAATTGAGTTCTGCTGAAGGATACTGGATCAGTGCTTTCGGGGGCTCTGTTG |
