# Supplementary material for: tRF-3005a regulates exon skipping of SPAG4 by interacting with RALY to drive gastric cancer progression
Source: Cell Death Discov. 2026 Mar 24;12:169. doi: 10.1038/s41420-026-03049-3 (PMC13039196; doi:10.1038/s41420-026-03049-3)
Supplement: Supplementary file 10 — Supplementary Table 3 [file 41420_2026_3049_MOESM10_ESM.docx]

**Supplementary Table 3 Sequences of the RNA pull-down probes.**

| **Oligonucleotide sequences of tRF-3005a used in the RNA pull-down (5’-3’, 3'Biotin).** |
| --- |
| **Sense**: UCUCGGUGGGACCUCCA |
| **Antisense**: UGGAGGUCCCACCGAGA |

| **Sequence of SPAG4 in pcDNA3.0 used in RNA pulldown assay (5’-3’).** |
| --- |
| GTGACGTCAGCAGCCGGCCGGGACACAGCGGGAGGGCAGGTGCGGCCGCGGGGCCTGCCGACTTCACGCAGGGTCCGTGGGGTCCCCGCGGCGCGCAGCGGCTGAAGGAGGCCCCAGGGCCTTGGCGACCGCAGCGGCGGCTTTAGCGTCAGTGACTAGGCAGCAGGGGGTCAGGATGCGGCGAAGCTCCCGCCCGGGCTCGGCCTCGTCCTCGCGCAAGCACACGCCCAACTTTTTCAGCGAGAACAGCTCAATGAGCATCACCTCGGAGGACAGCAAAGGGCTCCGGTCAGCGGAGCCCGGGCCTGGGGAGCCCGAGGGCAGAAGAGCCCGGGGCCCGAGCTGCGGTGAGCCCGCCTTGAGCGCGGGAGTGCCCGGAGGAACCACATGGGCAGGAAGCTCTCAGCAGAAGCCAGCGCCTCGGAGCCACAACTGGCAGACAGCCTGTGGCGCGGCAACCGTGAGGGGCGGGGCCTCGGAACCGACTGGGTCTCCAGTAGTCTCTGAGGAGCCGCTCGACCTTCTCCCGACCCTGGATCTGAGGCAGGAGATGCCTCCCCCGCGGGTGTTCAAGAGCTTTCTGAGCCTGCTCTTCCAGGGGCTGAGCGTGTTGTTATCCCTGGCAGGAGACGTGCTGGTCAGCATGTACAGGGAGGTCTGTTCCATCCGCTTCCTGTTCACGGCTGTGTCGCTGCTGAGCCTCTTTCTGTCAGCATTCTGGCTGGGGCTTCTGTACCTGGTCTCTCCTTTGGAGAATGAACCTAAGGAGATGCTGACTCTAAGTGAGTACCACGAGCGCGTGCGCTCCCAGGGGCAGCAGCTGCAGCAGCTCCAGGCCGAGCTGGATAAACTCCACAAGGAGGTGTCCACTGTTCGGGCAGCCAACAGCGAGAGAGTGGCCAAGCTCGTGTTCCAGAGGCTGAATGAGGATTTTGTGCGGAAGCCCGACTATGCTTTGAGCTCTGTGGGAGCCTCCATCGACCTGCAGAAGACATCCCACGATTACGCAGACAGGAACACTGCCTACTTCTGGAATCGCTTCAGCTTCTGGAACTACGCACGGCCGCCCACGGTTATCCTGGAGCCCCACGTGTTCCCTGGGAATTGCTGGGCTTTTGAAGGCGACCAAGGCCAGGTGGTGATCCAACTGCCGGGCCGAGTGCAGCTGAGCGACATCACTCTGCAGCATCCACCGCCCAGCGTGGAGCACACCGGAGGAGCCAACAGCGCCCCCCGCGATTTCGCGGTCTTTGGCCTCCAGGTTTATGATGAAACTGAAGTTTCCTTGGGGAAATTCACCTTCGATGTTGAGAAATCGGAGATTCAGACTTTCCACCTGCAGAATGACCCCCCAGCTGCCTTTCCCAAGGTGAAGATCCAGATTCTAAGCAACTGGGGCCACCCCCGTTTCACGTGCTTGTATCGAGTCCGTGCCCACGGTGTGCGAACCTCAGAGGGGGCAGAGGGCAGTGCACAGGGGCCCCATTAAACATGCTGATTTTTGGAGTAGAATTGAGTTCTGCTGAAGGATACTGGATCAGTGCTTTCGGGGGCTCTGTTG |

| **Oligonucleotide sequences of SPAG4 in pcDNA3.0 used in the RNA pulldown assay (5’-3’).** |
| --- |
| **Oligo1, intron7:**  GTGAGCCCCGGCCCACCTTGGAAACCCCTGATGGATCCCTGCTCCCAGGGTCCTGAGACTTAAGCTCCGCCCAGAGCCCTGCGGGATTTCTCCCGGTGCCCACGAAGTCCATCCCAAAGCAACCAGCTCCCAGAGGTCCCGTCCACCTGTAAAAGCAGCCCGCAAGCCTCGCCCCTCCAGGCCTCGCCCTCCCTCCTTGCAG |
| **Oligo2, exon7:**  TGAGTACCACGAGCGCGTGCGCTCCCAGGGGCAGCAGCTGCAGCAGCTCCAGGCCGAGCTGGATAAACTCCACAAGGAGGTGTCCACTGTTCGGGCAGCCAACAGCGAG |
| **Oligo3, intron8:**  GTAAGACCCGGAGACACTGGAAGACAGAGACGCAGACAGGAAAGAGGCCAAGACACTGACACAGACAGACCCATGCACCTGACCGGCCGAAGACAGAGCCTCGGACAGCCCCCACCCGCCCCCAGCCCCCGCGCCCCCGCGCCCCGACTCCCGGCAAGGCCTGGGAGCCTCTGAGGGTTACTTCTCACTGTTCCAG |
| **Oligo4, enon8:**  AGAGTGGCCAAGCTCGTGTTCCAGAGGCTGAATGAGGATTTTGTGCGGAAGCCCGACTATGCTTTGAGCTCTGTGG |
| **Oligo5, intron9:**  GTGAGTCTGGAAACATCCCGGGATGGGACCCCGGGCGGGACGCTGGGAAAAGCACCAAAACCCCGCCCTACAGGCGGAGCTTGGCTGAGCCGAGGGCGTGGAGGATGGCGTTTAACTCAAGGAGAGACCTGGCATTGCTGGGGACTGGGGCGGGCTGGAGGTGGAGCTCTGGGCGGGTCGATGGATGGGGTCGAGCTGAGGCCCGGGCTGGGGAGCGGCAGCAGTCGCTCTGTCCGACGGTTCCGATGGTCCCTCCGCCCGCCTGCAG |
