# Supplementary material for: tRF-3005a regulates exon skipping of SPAG4 by interacting with RALY to drive gastric cancer progression
Source: Cell Death Discov. 2026 Mar 24;12:169. doi: 10.1038/s41420-026-03049-3 (PMC13039196; doi:10.1038/s41420-026-03049-3)
Supplement: Supplementary file 11 — Supplementary Table 4 [file 41420_2026_3049_MOESM11_ESM.docx]

Supplementary Table 4 Sequences of the FISH probes.

| Groups | Sequences (5’ to 3’) |
| --- | --- |
| tRF-3005a | TGGAGGTCCCACCGAGA |
| U6 | TTTGCGTGTCATCCTTGCG |
| 18S | CTTCCTTGGATGTGGAGCCGTTTC |
