# Supplementary material for: tRF-3005a regulates exon skipping of SPAG4 by interacting with RALY to drive gastric cancer progression
Source: Cell Death Discov. 2026 Mar 24;12:169. doi: 10.1038/s41420-026-03049-3 (PMC13039196; doi:10.1038/s41420-026-03049-3)
Supplement: Supplementary file 12 — Supplementary Table 5 [file 41420_2026_3049_MOESM12_ESM.docx]

Supplementary Table 5:

The 81 specific tRF-3005a binding proteins identified by mass spectrometry are shown.

| Accession | Genes | Coverage (%) | PSMs | Unique Peptides | Molecular Weight (kDa) | calc. pI |
| --- | --- | --- | --- | --- | --- | --- |
| A6NMY6 | ANXA2P2 | 8.00 | 4 | 4 | 38.6 | 6.95 |
| P16989 | YBX3 | 7.00 | 2 | 2 | 40.1 | 9.77 |
| P12236 | SLC25A6 | 6.00 | 2 | 1 | 32.8 | 9.74 |
| P78406 | RAE1 | 7.00 | 2 | 2 | 40.9 | 7.83 |
| Q5T749 | KPRP | 3.00 | 3 | 2 | 64.1 | 8.27 |
| Q14103 | HNRNPD | 7.00 | 2 | 2 | 38.4 | 7.81 |
| P46940 | IQGAP1 | 2.00 | 3 | 3 | 189.1 | 6.48 |
| P61626 | LYZ | 8.00 | 1 | 1 | 16.5 | 9.16 |
| P05198 | EIF2S1 | 6.00 | 2 | 2 | 36.1 | 5.08 |
| O00571 | DDX3X | 3.00 | 2 | 2 | 73.2 | 7.18 |
| Q08945 | SSRP1 | 2.00 | 1 | 1 | 81.0 | 6.87 |
| P35579 | MYH9 | 1.00 | 1 | 1 | 226.4 | 5.60 |
| P12956 | XRCC6 | 4.00 | 2 | 2 | 69.8 | 6.64 |
| P29508 | SERPINB3 | 4.00 | 2 | 2 | 44.5 | 6.81 |
| Q9Y446 | PKP3 | 2.00 | 1 | 1 | 87.0 | 9.32 |
| O00303 | EIF3F | 5.00 | 1 | 1 | 37.5 | 5.45 |
| O43242 | PSMD3 | 4.00 | 2 | 2 | 60.9 | 8.44 |
| Q9Y3I0 | RTCB | 2.00 | 1 | 1 | 55.2 | 7.23 |
| P0DME0 | SETSIP | 3.00 | 1 | 1 | 34.9 | 4.31 |
| P0DP25 | CALM3 | 9.00 | 1 | 1 | 16.8 | 4.22 |
| Q86V81 | ALYREF | 4.00 | 1 | 1 | 26.9 | 11.15 |
| P11208 | P/V | 4.00 | 1 | 1 | 42.1 | 8.66 |
| P17987 | TCP1 | 2.00 | 1 | 1 | 60.3 | 6.11 |
| Q9BYJ9 | YTHDF1 | 3.00 | 2 | 2 | 60.8 | 8.79 |
| P39023 | RPL3 | 3.00 | 2 | 2 | 46.1 | 10.18 |
| Q6NXG1 | ESRP1 | 2.00 | 1 | 1 | 75.5 | 6.68 |
| Q13085 | ACACA | 0.00 | 1 | 1 | 265.4 | 6.37 |
| P13010 | XRCC5 | 1.00 | 1 | 1 | 82.7 | 5.81 |
| O75643 | SNRNP200 | 0.00 | 1 | 1 | 244.4 | 6.06 |
| O60832 | DKC1 | 2.00 | 1 | 1 | 57.6 | 9.42 |
| Q03252 | LMNB2 | 2.00 | 1 | 1 | 69.9 | 5.59 |
| P25788 | PSMA3 | 5.00 | 1 | 1 | 28.4 | 5.33 |
| P48643 | CCT5 | 2.00 | 1 | 1 | 59.6 | 5.66 |
| P38919 | EIF4A3 | 3.00 | 1 | 1 | 46.8 | 6.73 |
| P78527 | PRKDC | 0.00 | 1 | 1 | 468.8 | 7.12 |
| P38646 | HSPA9 | 2.00 | 1 | 1 | 73.6 | 6.16 |
| Q9H9B4 | SFXN1 | 4.00 | 1 | 1 | 35.6 | 9.07 |
| Q92804 | TAF15 | 2.00 | 1 | 1 | 61.8 | 8.02 |
| P15311 | EZR | 1.00 | 1 | 1 | 69.4 | 6.27 |
| O76094 | SRP72 | 1.00 | 1 | 1 | 74.6 | 9.26 |
| P35658 | NUP214 | 0.00 | 1 | 1 | 213.5 | 7.47 |
| Q13162 | PRDX4 | 3.00 | 1 | 1 | 30.5 | 6.29 |
| Q99848 | EBNA1BP2 | 2.00 | 1 | 1 | 34.8 | 10.10 |
| P26599 | PTBP1 | 2.00 | 1 | 1 | 57.2 | 9.17 |
| O75746 | SLC25A12 | 1.00 | 1 | 1 | 74.7 | 8.38 |
| P26368 | U2AF2 | 2.00 | 1 | 1 | 53.5 | 9.09 |
| P28072 | PSMB6 | 5.00 | 1 | 1 | 25.3 | 4.92 |
| Q86UE4 | MTDH | 2.00 | 1 | 1 | 63.8 | 9.32 |
| Q86VP6 | CAND1 | 1.00 | 1 | 1 | 136.3 | 5.78 |
| O43707 | ACTN4 | 1.00 | 1 | 1 | 104.8 | 5.44 |
| Q9UHB9 | SRP68 | 1.00 | 1 | 1 | 70.7 | 8.56 |
| Q9Y224 | RTRAF | 4.00 | 1 | 1 | 28.1 | 6.65 |
| Q13835 | PKP1 | 1.00 | 1 | 1 | 82.8 | 9.13 |
| B5ME19 | EIF3CL | 1.00 | 1 | 1 | 105.4 | 5.64 |
| P41252 | IARS1 | 1.00 | 1 | 1 | 144.4 | 6.15 |
| P25789 | PSMA4 | 3.00 | 1 | 1 | 29.5 | 7.72 |
| P49756 | RBM25 | 1.00 | 1 | 1 | 100.1 | 6.32 |
| P68400 | CSNK2A1 | 2.00 | 1 | 1 | 45.1 | 7.74 |
| Q9UH65 | SWAP70 | 2.00 | 1 | 1 | 69.0 | 5.87 |
| Q9UJV9 | DDX41 | 1.00 | 1 | 1 | 69.8 | 6.84 |
| P08621 | SNRNP70 | 2.00 | 1 | 1 | 51.5 | 9.94 |
| P14868 | DARS1 | 2.00 | 1 | 1 | 57.1 | 6.55 |
| Q09028 | RBBP4 | 2.00 | 1 | 1 | 47.6 | 4.89 |
| Q9Y520 | PRRC2C | 0.00 | 1 | 1 | 316.7 | 9.13 |
| P07814 | EPRS1 | 1.00 | 1 | 1 | 170.5 | 7.33 |
| P50991 | CCT4 | 1.00 | 1 | 1 | 57.9 | 7.83 |
| P35606 | COPB2 | 1.00 | 1 | 1 | 102.4 | 5.27 |
| Q9UKM9 | RALY | 3.00 | 1 | 1 | 32.4 | 9.17 |
| Q12849 | GRSF1 | 2.00 | 1 | 1 | 53.1 | 6.19 |
| O15303 | GRM6 | 2.00 | 1 | 1 | 95.4 | 8.02 |
| Q9BYK8 | HELZ2 | 0.00 | 1 | 1 | 294.5 | 7.49 |
| Q9UHX1 | PUF60 | 1.00 | 1 | 1 | 59.8 | 5.29 |
| Q99567 | NUP88 | 1.00 | 1 | 1 | 83.5 | 5.69 |
| Q8IZW8 | TNS4 | 1.00 | 1 | 1 | 76.7 | 7.34 |
| P46087 | NOP2 | 1.00 | 1 | 1 | 89.2 | 9.23 |
| O75400 | PRPF40A | 1.00 | 1 | 1 | 108.7 | 7.56 |
| P26196 | DDX6 | 2.00 | 1 | 1 | 54.4 | 8.66 |
| Q9BZK7 | TBL1XR1 | 4.00 | 1 | 1 | 55.6 | 5.55 |
| Q8NE71 | ABCF1 | 1.00 | 1 | 1 | 95.9 | 6.80 |
| P04844 | RPN2 | 1.00 | 1 | 1 | 69.2 | 5.69 |
| Q9UPT8 | ZC3H4 | 3.00 | 1 | 1 | 140.2 | 6.27 |
