# Supplementary material for: tRF-3005a regulates exon skipping of SPAG4 by interacting with RALY to drive gastric cancer progression
Source: Cell Death Discov. 2026 Mar 24;12:169. doi: 10.1038/s41420-026-03049-3 (PMC13039196; doi:10.1038/s41420-026-03049-3)
Supplement: Supplementary file 13 — Supplementary Table 6 [file 41420_2026_3049_MOESM13_ESM.docx]

**Supplementary Table 6 Downstream differentially expressed genes of SPAG4 in tRF-3005a inhibition cells**

| Gene Symbol | foldchange | Regulation | P-value |
| --- | --- | --- | --- |
| RNU6-1 | 0.002569 | Down | 2.76E-10 |
| GRB14 | 0.201168 | Down | 0.000126 |
| MEOX1 | 0.322504 | Down | 1.95E-06 |
| RPS24P17 | 0.377136 | Down | 9.75E-10 |
| ACSM3 | 0.377923 | Down | 0.001049 |
| CDH5 | 0.399631 | Down | 6.34E-07 |
| PLCH1 | 0.401508 | Down | 0.000463 |
| RGS3 | 0.449927 | Down | 5.36E-06 |
| RFX3 | 0.474129 | Down | 1.10E-07 |
| ACACA | 0.496677 | Down | 2.95E-30 |
